# Wavelet neural network based reduced-ripple DITC of switched reluctance motors in electric vehicles

**DOI:** 10.1038/s41598-026-46371-7

**Published:** 2026-03-31

**Authors:** Ameer L. Saleh, Mahmoud Hamouda, László Számel, Mohammad A. Abido

**Affiliations:** 1 Department of Electric Power Engineering, Budapest University of Technology and Economics, Budapest, 1111 Hungary; 2 Department of Electrical Engineering, University of Misan, Misan, 62001 Iraq; 3 Interdisciplinary Research Center for Sustainable Energy Systems (IRC-SES), KFUPM, Dhahran, Saudi Arabia; 4 Electrical Engineering Department, KFUPM, Dhahran, Saudi Arabia

**Keywords:** Switched reluctance motors, Direct instantaneous torque control (DITC), Wavelet neural network (WNN), Electric vehicles (EVs), Equilibrium optimizer (EO), Energy science and technology, Engineering

## Abstract

Switched Reluctance Motors (SRMs) are potential candidates for high-performance and cost-effective electric drives owing to their simple structure, robustness, fault-tolerant control, high-reliability, and high-efficiency. However, their highly nonlinear magnetic characteristics and doubly salient structure inherently generate considerable torque ripples, which limit their widespread adoption across various industrial applications. This paper introduces an enhanced Direct Instantaneous Torque Control (DITC) scheme augmented with a Wavelet Neural Network (WNN) to minimize torque ripples. The proposed WNN is employed to dynamically compensate for the torque error, hence adjusting the reference torque signal and providing an optimal input for the hysteresis torque controller. This nonlinear mechanism can significantly mitigate the torque ripples, enhance the quality of torque profiles, and maintain the required average torque. A fixed-gain, 3 layers, 2 Neurons, 7 parameters, single WNN is implemented for compensating the torque error, considering the different operating conditions; the Equilibrium Optimizer (EO) algorithm is utilized to train and optimize the network parameters (weights, translation, and dilation of wavelet functions). The simulation and experimental results confirm the superior performance of the proposed DITC-WNN compared to conventional schemes. The proposed DITC-WNN shows experimental reductions in torque ripples of about 28.8% for heavy loads and 16% for light loads over a wide speed range, confirming its suitability for high-performance and reduced ripple SRM drives.

## Introduction

Switched Reluctance Motors (SRMs) are competitive candidates for a variety of industrial applications, including electric vehicles (EVs), owing to their attractive merits such as simple and robust structure, low manufacturing and maintenance costs, and the absence of rare-earth materials^1,2^. Though the high torque ripples are the inherent drawback of SRMs, restricting their adoption in high-performance applications^3,4^.

Over the past decades, substantial research efforts have been devoted to enhancing the performance of SRM drives by optimizing machine structure, refining magnetic models, and developing advanced control strategies^5,6^. Even though the machine design optimization can mitigate torque ripple to some extent, its effectiveness is constrained^7^. Alternatively, the control strategies have proven more effectiveness by reducing the torque ripples and improving the dynamic performance^4,8^. Traditional current control approaches, such as current chopping control (CCC), are simple and cost-effective but inherently generate torque ripples due to their dependence on a square-wave current regulation^9,10^. This limitation highlights the necessity of torque-based control approaches. The torque control approach demonstrates increased efficacy and potential for addressing torque ripple problems compared with current control. Among torque control strategies, average torque control (ATC), instantaneous torque control (ITC), and model predictive torque control (MPTC) are widely studied^11–13^.

The ATC scheme merits the simple control structure and wide operational range of speeds, and encounters the relatively high torque ripples. The MPTC scheme exhibits an outstanding dynamic response by employing a predictive model to forecast the system’s future behavior and select appropriate control actions^14^; it effectively handles real-time parameter variations in SRMs. However, its performance depends mainly on the accuracy of its prediction models, facing serious challenges to consider the highly nonlinear magnetic characteristics of SRMs, some suppositions are often required that may impact the performance^15^. Conversely, the Instantaneous Torque Control (ITC) strategies have inherited the capability to reduce the torque ripples in SRMs, involving the Indirect Instantaneous Torque Control (IITC) based on the torque sharing function (TSF) and the Direct Instantaneous Torque Control (DITC). The IITC based TSFs involve an indirect torque control strategy by regulating the currents; a torque-to-current conversion is then essential^16^. However, the real-time implementation of an accurate torque-to-current conversion complicates the control algorithm and affects its control performance. Conversely, the DITC provides a simplified control structure by eliminating torque-to-current conversion; it controls directly the instantaneous motor torque using a hysteresis torque controller^17^. Nevertheless, traditional DITC still encounters issues with negative torque and considerable torque ripples^18^. To overcome these drawbacks of DITC, several strategies have been proposed recently. In^19^, a new adaptive dynamic commutation is introduced to promote the performance of DITC by mitigating its torque ripple and improving the efficiency. The adaptive commutation mechanism in the proposed scheme achieves good torque tracking by dynamically adjusting the commutation regions; online switching angles are modified according to the instantaneous torque error and phase current endpoint detection, resulting in minimizing the torque ripple and boosting energy utilization. In^20^, an improved DITC strategy is introduced based on switch-on angle optimization and adaptive modification of the conduction regions corresponding to the inductance change rate. The turn-on angle had optimized online employing an upgraded Generalized Regression Neural Network (GRNN). In^21^, a modified DITC based on an online optimization approach for switching angles was suggested to suppress torque ripple and boost the efficiency of SRMs. The online optimization algorithm of switch-on and switch-off angles was developed according to the inductance and flux linkage characteristics, respectively. Then, the torque generation ability is maximized by adopting the conduction rules in different regions. In^22^, an advanced DITC scheme for SRMs using a boosting chopper converter and a dual-PI control method is introduced to extend voltage utilization and improve torque profile accuracy during transient states. The proposed strategy predicts the dc-link voltage and distributes the torque during the commutation region to mitigate torque ripple during the commutation period and to enhance the motor efficiency. In^23^, an enhanced DITC incorporating optimized TSF was developed using the Grey Wolf Optimization (GWO) algorithm, with the objective of reducing and improving efficiency; it integrated the fast dynamic response of DITC with the torque-smoothing capability of TSF, ensuring a balanced phase torque distribution and thereby maintaining a constant instantaneous total torque. Similarly, in^24^, a vehicle-mounted DITC strategy incorporating an optimized TSF is developed to get a better torque profile and improve the drive performance during dynamic operating conditions. This approach reshapes the TSF by optimizing the commutation angles utilizing an artificial bee colony (ABC) algorithm, thereby generating the optimal torque references for each phase. Furthermore, nonlinear control-based DITC strategies, incorporating a sliding mode controller, have also been suggested in^25–27^. In^25^, a hybrid control scheme that incorporates nonsingular fast terminal sliding mode control (NFTSMC) with a DITC strategy was presented for a six-phase SRM to suppress torque ripple and improve the dynamic response. The proposed strategy employed modified dynamic conduction rules to enhance the effectiveness of torque control in DITC, particularly in high-speed operating conditions. Moreover, an NFTSMC controller based on a developed approach law is employed to effectively accelerate the response time and decrease chattering. In^26^, the performance of DITC is improved by employing a terminal sliding mode scheme-based speed controller. The control parameters are optimized using a hybrid GWO algorithm. Similarly, in^27^, a modified SMC-based speed controller with a disturbance observer is introduced to suppress torque ripple and improve dynamic response. On the contrary, adaptive PWM strategies can improve the performance of DITC^28,29^. In^28^, a hysteresis segmented PWM-DITC strategy was proposed to effectively solve the issue of high torque ripple during commutation caused by unsmooth hysteresis control. In^29^, a hysteresis segmented PWM-DITC and zero-voltage modulation strategies for permanent magnet–assisted SRMs are presented to enhance torque continuity and decrease switching losses. The proposed strategy divided the control intervals according to inductance and torque-to-current ratio (TCR), with online adjustment of the duty cycle to improve torque tracking and suppress torque ripples. Furthermore, the switching angles are optimized by the dung-beetle-optimized back propagation (BP) neural network (DBO-BP) algorithm, which mitigates the torque ripple, lowers phase current peaks, and boosts motor efficiency. Recently, intelligent control techniques have been incorporated into the DITC strategy to achieve optimal switching logic and adaptive torque regulation^20,30^. In^30^, an enhanced DITC strategy employed an artificial neural network (ANN) based on a modified PSO algorithm that was presented to optimize the switch-off angle online under different operating conditions to mitigate the generating negative torque. While the switch-on angle is dynamically adjusted based on flux theoretical derivation to enable precise torque tracking.

To overcome the limitations of conventional DITC, this paper proposes a novel Wavelet Neural Network based enhanced DITC strategy for SRMs. The main contributions of this paper are as follows:

1. An optimized Wavelet Neural Network (WNN) is proposed, designed, and implemented for the online compensation of the reference torque signal, to improve the torque profile and minimize torque ripples.
2. A simplified optimization procedure is developed to train the proposed WNN. A fixed-gain, 3-layer, 7-parameter WNN is achieved for compensating the reference torque signal, considering the different operating conditions; the Equilibrium Optimizer (EO) algorithm is utilized to train and optimize the 7 parameters (weights, translation, and dilation of wavelet functions) of the proposed WNN.
3. The integrated optimized DITC–WNN scheme effectively addresses the primary shortcomings of existing DITC techniques by combining adaptive learning, global optimization, and instantaneous torque regulation for a high-performance SRM drive. The torque ripples during phase overlap and commutation are significantly minimized through the WNN’s real-time adaptive torque shaping, providing a simple and generalized torque control strategy for several industrial applications involving EVs.

The remainder of this paper is formed as follows: Section II discusses the proposed DITC–WNN. Sections III and IV include the simulation and experimental results, respectively. Section V is the conclusion.

## The proposed DITC based wavelet neural network (DITC–WNN)

**Fig. 1:**
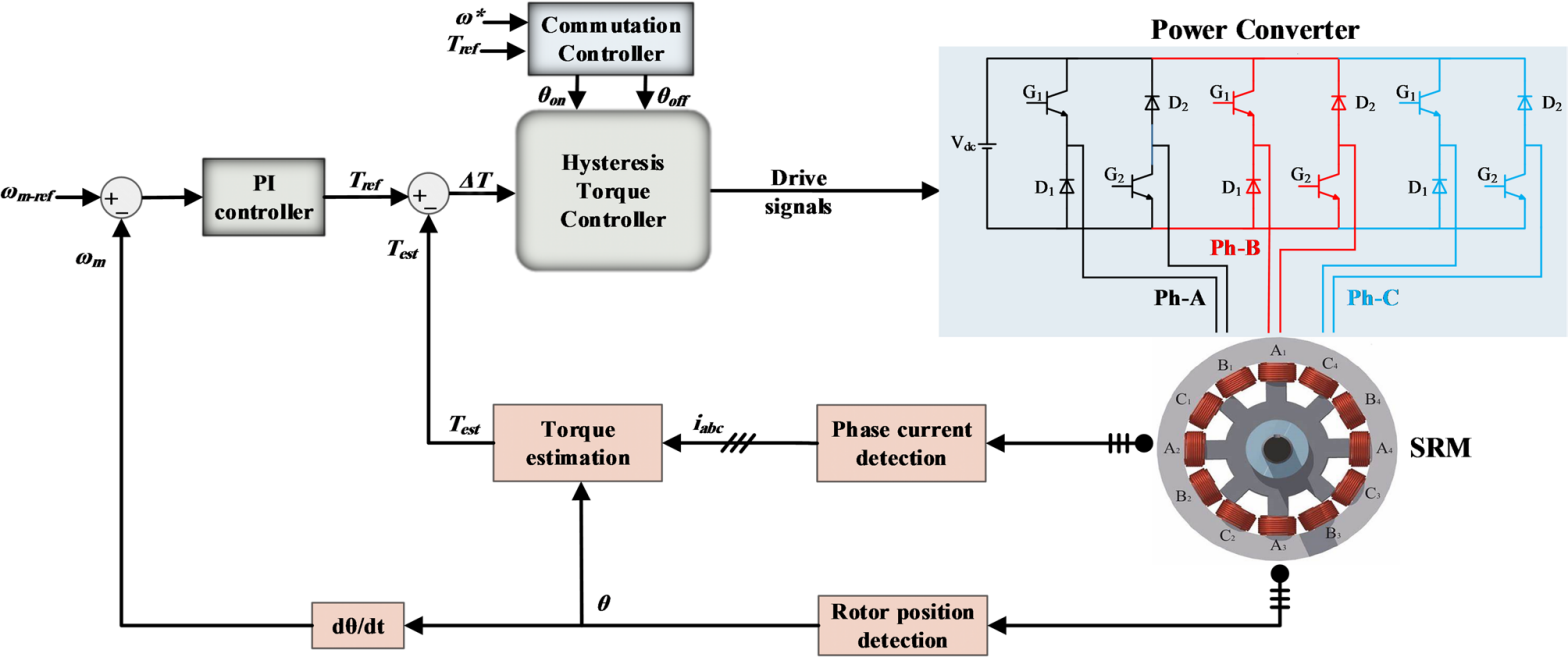
Conventional DITC strategy for SRM drive system.

First, Fig. 1 depicts the block diagram of standard DITC; the outer-loop speed controller generates the reference torque signal (*T*_*ref*_); the instantaneous torque (*T*_*est*_) is estimated online based on the measured data of phases’ currents and position. The resulting torque error (*ΔT*=*T*_*ref*_ −*T*_*est*_ ) serves as the control input for the hysteresis torque controller (HTC). According to the torque error (*ΔT*), the HTC generates the drive signals for the asymmetric half-bridge converter (AHBC). The DITC offers a simple control structure, addressing the challenges associated with IITC schemes, such as the requirement for torque-to-current conversion and closed-loop current control^19^.

**Fig. 2:**
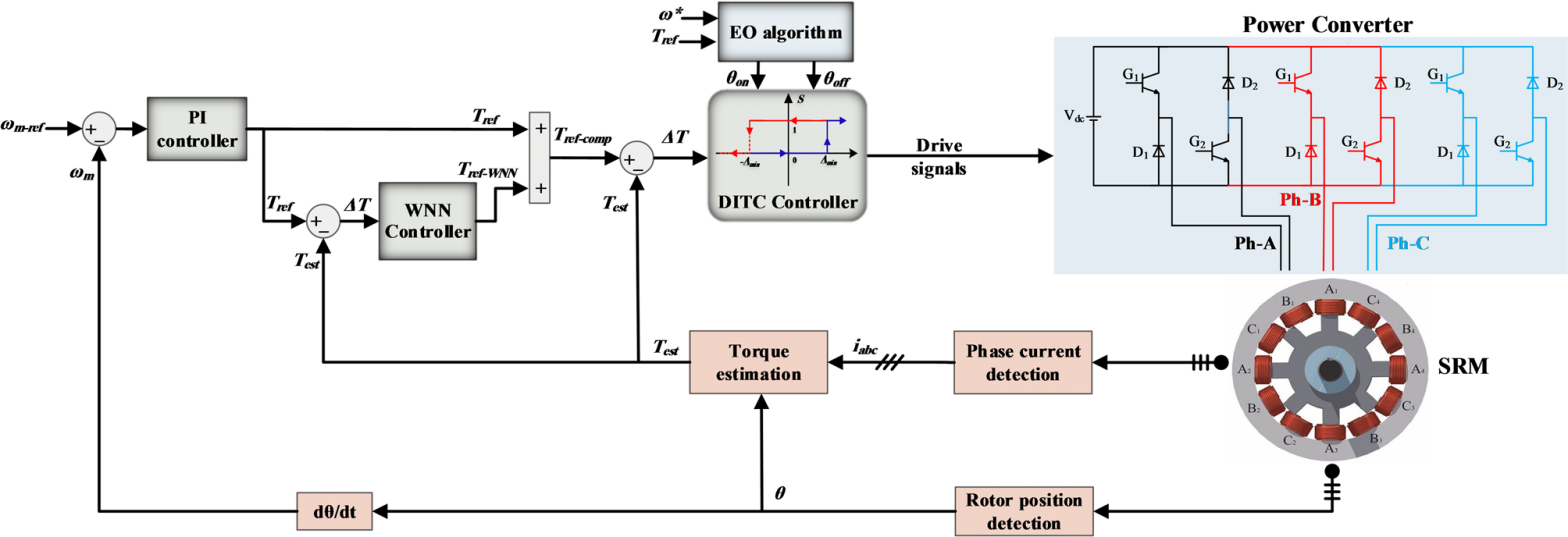
Proposed DITC strategy based on adaptive WNN.

To address the issues of torque ripples in the commutation regions resulting from the single control strategy used in the conventional DITC, this work proposes a novel enhanced DITC strategy based on an optimized WNN. The proposed DITC-WNN scheme is presented in Fig. 2; the WNN is placed between the outer loop speed controller and the HTC, its inputs are the torque error (*ΔT*) and its derivative (*ΔT’*), and it outputs the compensated reference torque signal.

The objective of WNN is to generate a nonlinear reference torque signal based on the real operating conditions, rather than applying a fixed torque reference directly to the HTC. This modified approach enhances the dynamic performance of HTC by minimizing the torque ripples over a wide range of operating scenarios. The WNN operates as a nonlinear torque reference generator based on the torque error and its derivative signals. The parameters of WNN are trained offline using the EO algorithm^31^ to ensure fast learning and adequate generalization capability, the design and training details of the proposed WNN are given as follows:

### The proposed wavelet neural network

The WNNs can be classified as an advanced architecture class of neural networks, integrating the wavelet theory with the conventional architectures of neural networks. Within the hidden layer, the wavelet function is used as an activation function, replacing the commonly used sigmoid or radial basis functions in standard neural networks. These integrations power the ability of WNNs to approximate complex, nonlinear, and time-varying functions with high precision and adaptability^32^. The WNN acts as a nonlinear compensator to learn position-dependent torque profile components of SRM by exploiting the time-frequency localization ability of wavelets which leads a low torque ripple and high-performance system.

**Fig. 3:**
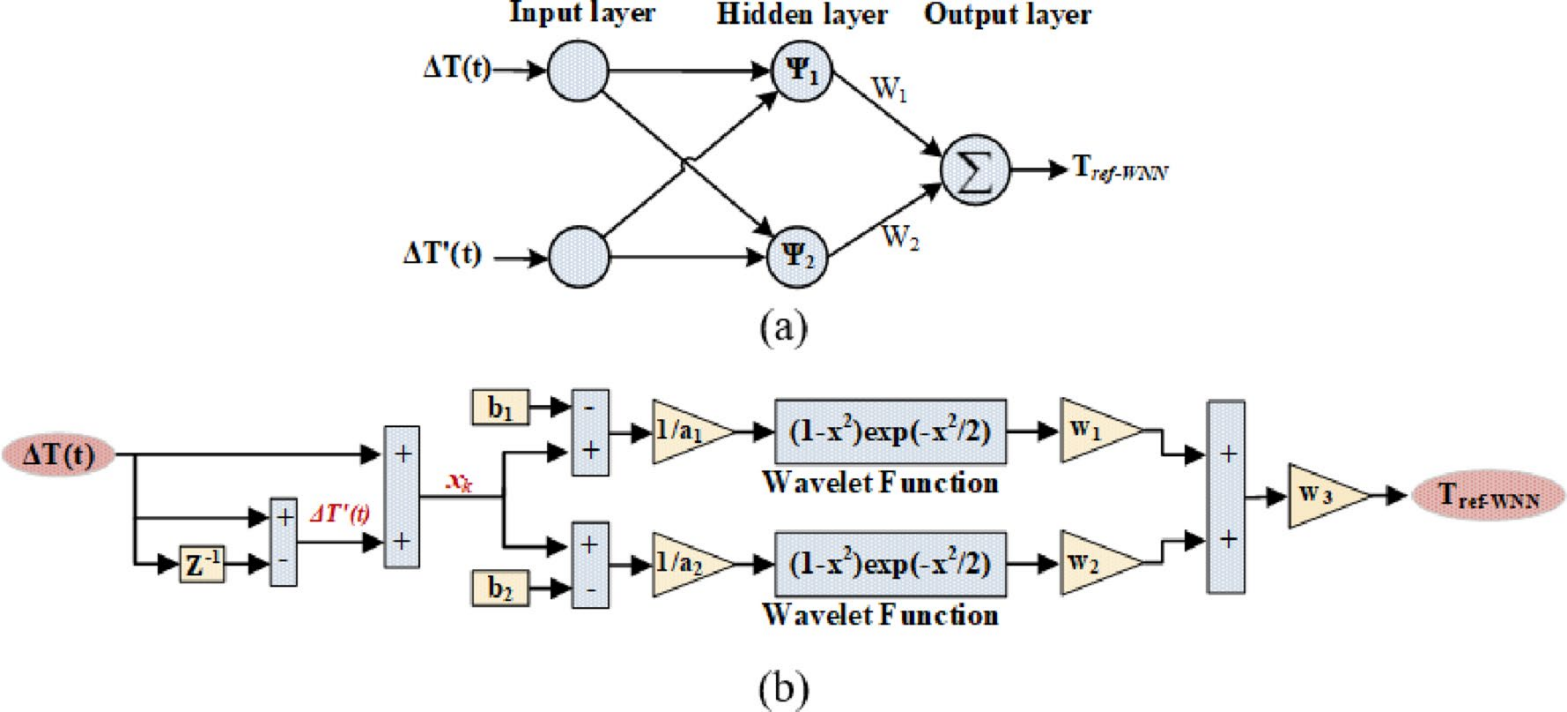
The structure of WNN control (**a**) 3-layer structure of WNN, (**b**) MATLAB Simulink model of WNN.

Figure 3 exhibits the structural configuration of the proposed WNN, consisting of three layers: an input layer, a hidden wavelet layer, and an output layer.

- The input layer receives both the torque error (*ΔT*) and its derivative (*ΔT’*).
- The hidden layer has 2 neurons; it employs the Mexican Hat wavelet as the activation function. Each neuron is characterized by an adaptive dilation parameter (a_k_) and a translation parameter (b_k)_, which enable flexible scaling and shifting of the wavelet basis functions. Noting that number of hidden neurons was selected through a parametric evaluation of several WNN configurations during the preliminary design stage. Shallow (one hidden layer with two neurons) and deeper structures (one hidden layer with 5–7 neurons) were assessed based on torque ripple reduction, peak current limitation, convergence behavior, and computational complexity. The results showed that increasing the number of neurons beyond two yielded only marginal improvements in torque ripple reduction while significantly increasing computational cost and execution time. In contrast, the two-neuron structure achieved satisfactory torque ripple mitigation and peak current suppression with minimal complexity. Given the real-time implementation requirements of SRM drives, the selected configuration provides an effective trade-off between control performance and computational efficiency.
- The output layer performs a weighted summation of the hidden neuron outputs to generate the nonlinear compensation control signal.

The mathematical formulation is as follows^33^:

Input Layer: This layer receives two inputs (ΔT(t), ΔT‘(t)) at time *t*, which can be stated as follows:

1 $$x_k(t) = \Delta T(t) + \dot{\Delta T}(t)$$

Hidden layer: The inputs to each hidden layer at time instant t, can be indicated as:

2 $$\:{\phi\:}_{k}\left(\mathrm{t}\right)={\uppsi\:}\left(\frac{{x}_{k}\left(\mathrm{t}\right)-{\mathrm{b}}_{\mathrm{k}}}{{\mathrm{a}}_{\mathrm{k}}}\right)$$

Accordingly, the output for each neuron layer is computed based on a Mexican Hat wavelet function as follows:

3 $$\:{{\uppsi\:}}_{k}\left(\mathrm{t}\right)=\left(1-{\phi\:\left(\mathrm{t}\right)}^{2}\right)\:{\mathrm{e}\mathrm{x}\mathrm{p}}^{\frac{{-\phi\:\left(\mathrm{t}\right)}^{2}}{2}}$$

Output layer: Finally, the output of the WNN is given as follows:

4 $$\:{T}_{ref-WNN}={w}_{3}\boldsymbol{*}\sum\:_{\mathrm{k}=1}^{m}{\mathrm{w}}_{k}{\uppsi\:}\left(\frac{{x}_{k}\left(\mathrm{t}\right)-{\mathrm{b}}_{\mathrm{k}}}{{\mathrm{a}}_{\mathrm{k}}}\right)$$

where *k* is the number of neurons in the hidden layer, *w*_*k*_ is the output weight.

### The proposed optimization procedure to train WNN

In this work, the EO is used to optimize the parameters of the proposed WNN utilizing a multi-objective function as depicted by (5), to suppress the torque ripples and to minimize torque errors.

5 $$\:{F}_{obj}\left({a}_{1},{a}_{2},{b}_{1},{b}_{2},{w}_{1},{w}_{2},{w}_{3}\right)={min}\left({w}_{r}\frac{{T}_{r}}{{T}_{r-b}}+{w}_{e}{\:e}_{t}\right)\:; \:{\:{w}_{r}+\:w}_{e}=1$$

where *F*_*obj*_, is the objective function value. *w*_*r*_ = 0.6 and *w*_*e*_= 0.4 are weighing factors for torque ripple and torque error, respectively. *T*_*r−b*_ is the base value of *T*_*r*_. While the integral of squared torque error (ISE) criterion can be described by:

6 $$\:{\:e}_{t}=\int\:{{\Delta\:}\mathrm{T}\left(\mathrm{t}\right)}^{2}\:\mathrm{d}\mathrm{t}$$

**Fig. 4:**
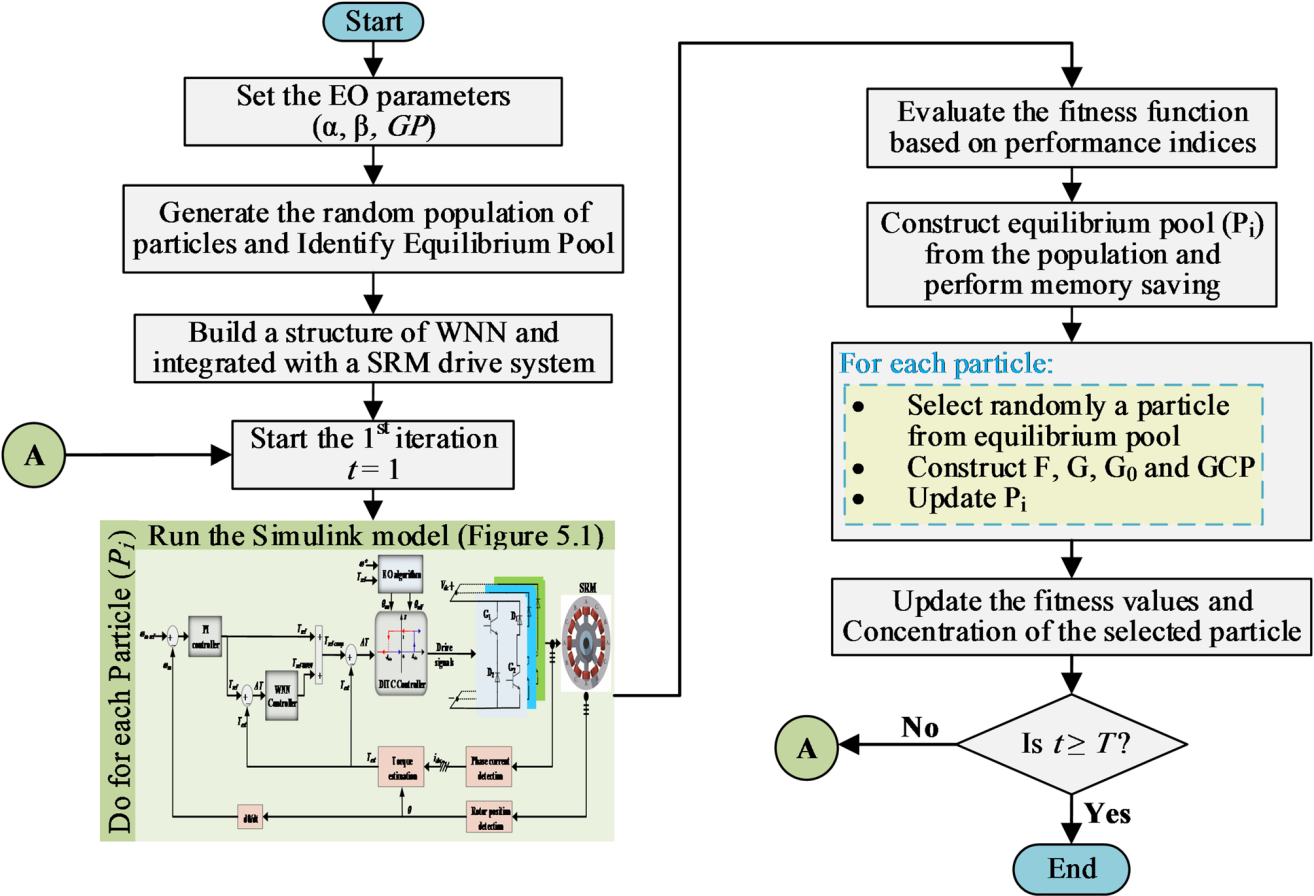
Flowchart of WNN-DITC-EO algorithm.

The training process of the proposed WNN is presented in Fig. 4. The algorithm begins by incorporating DITC-WNN into the SRM drive. Then, the EO is linked online to the WNN through the objective function in (5), allowing it to iteratively adjust the network parameters and find the optimal network output that achieves the lowest objective function.

In this work, the main contribution is to simplify the training procedure of the proposed WNN, hence reducing the training effort and time by providing a simple architecture control algorithm with fixed gains. By other means, the objective is to provide only 7 parameters ($\:{a}_{1},{a}_{2},{b}_{1},{b}_{2},{w}_{1},{w}_{2},{w}_{3}$) that can provide the control objective of reduced torque ripples over the different/complete operating conditions. There is no need to complicate the control algorithm with different sets of parameters ($\:{a}_{1},{a}_{2},{b}_{1},{b}_{2},{w}_{1},{w}_{2},{w}_{3}$) that can vary according to operating speed range and loading torque.

The proposed simplified training procedure is suggested to train the network parameters only once and only at one operating point; hence the obtained parameters of WNN are generalized for the complete drive system involving the different operating points of torque and speeds.

The question is which operating point that has this advantage to be generalized representing the complete motor drive; the answer is the high ripple points; any operating point that has a significant torque ripple, or significant torque error, is the best to be generalized representing the complete motor drive. The reason is that the proposed WNN processes the torque error signal and outputs a corresponding compensation for reference torque. So, to have a well-trained WNN, the inputted torque error must be noticeable in order to be controlled; that is the reason for considering only the operating points that have high torque ripple, which means also high torque errors. Noting that the chosen point should not be in the high-speed regions where the torque control is ineffective, it should be in the speed range where the motor current and hence torque are controlled to provide better torque profiles; high speeds, considering the SPC scheme, are out of interest.

This paper considers the operating point at a speed of 1000 r/min (rated speed is 800 r/min) and rated loading torque of 4 Nm; the multi-objective EO algorithm is employed to optimize and train the WNN parameters using data obtained from this single operating point (1000 r/min and 4 Nm). This procedure yields a simple WNN network that contains a compact set of 7 parameters capable of ensuring accurate torque control across the full range of speed and load conditions. The selection of operating point (1000 r/min and 4 Nm) is intentional and based on the following considerations:

1. This operating condition represents a high-speed, heavy-load region, where torque ripple is relatively high due to magnetic nonlinearity and commutation effects.
2. At this point, the system still maintains stable and accurate current control, ensuring that the observed torque ripple mainly reflects intrinsic electromagnetic characteristics rather than current regulation instability.
3. The combination of high speed and rated torque produces a wide torque ripple bandwidth, allowing the WNN to learn sufficient nonlinear features of the torque compensation mapping.
4. Compared to other high-ripple points (e.g., low-speed high-load or very high-speed light-load conditions), this operating point provides a balanced scenario where both ripple amplitude and dynamic behavior are representative of typical SRM operation.

Because it contains significant nonlinear ripple characteristics while remaining dynamically stable, this point serves as a representative training condition that enables effective generalization across different speed and load states.

The output optimization results for the network parameters are given below:

**Table:** 

Operating point	${a}_{1}$	${a}_{2}$	${b}_{1}$	${b}_{2}$	${w}_{1}$	${w}_{2}$	${w}_{3}$
1000 r/min, 4Nm	− 25	16.2	16.65	30.3	4.54	6.6545	12.89

### Optimized switching angles for proposed DITC approach

To improve the performance of the proposed DITC, the switching angles are optimized through the Equilibrium Optimizer (EO) algorithm based on a multi-objective function as demonstrated in Eq. (7); it prioritizes minimizing both the torque ripple and copper losses simultaneously^23^.

7 $$\:{F}_{obj}\left({\boldsymbol{\theta\:}}_{\boldsymbol{o}\boldsymbol{n}}{,\boldsymbol{\theta\:}}_{\boldsymbol{o}\boldsymbol{f}\boldsymbol{f}}\right)={min}\left({w}_{r}\frac{{T}_{r}}{{T}_{r\_b}}+{w}_{cu}\frac{{P}_{cu}}{{P}_{cu-b}}\right)\:; \:{\:{w}_{r}+\:w}_{cu}=1$$

For 12/8 SRM, the optimization problem is subjected to:

8 $$\:{-\theta\:}_{m}\le\:{\theta\:}_{on}\le\:{\theta\:}_{m};$$

9 $$\:{\theta\:}_{on}+{15}^{\circ\:}\le\:{\theta\:}_{off}\le\:{\theta\:}_{off}^{max}$$

In this work, higher priority is set for torque ripple with a weighting factor of *w*_*r*_ = 0.7; and a weighting factor of *w*_*cu*_ = 0.3 for the copper losses. $\:{\theta\:}_{off}^{max}$ is 22.5° for 12/8 SRM. While *T*_*r_b*_ and *P*_*cu-b*_ are the base values of *T*_*r*_ and *P*_*cu*_ respectively. $\:{\theta\:}_{m}$ is the angle at which rotor poles start to overlap with stator poles.

**Fig. 5:**
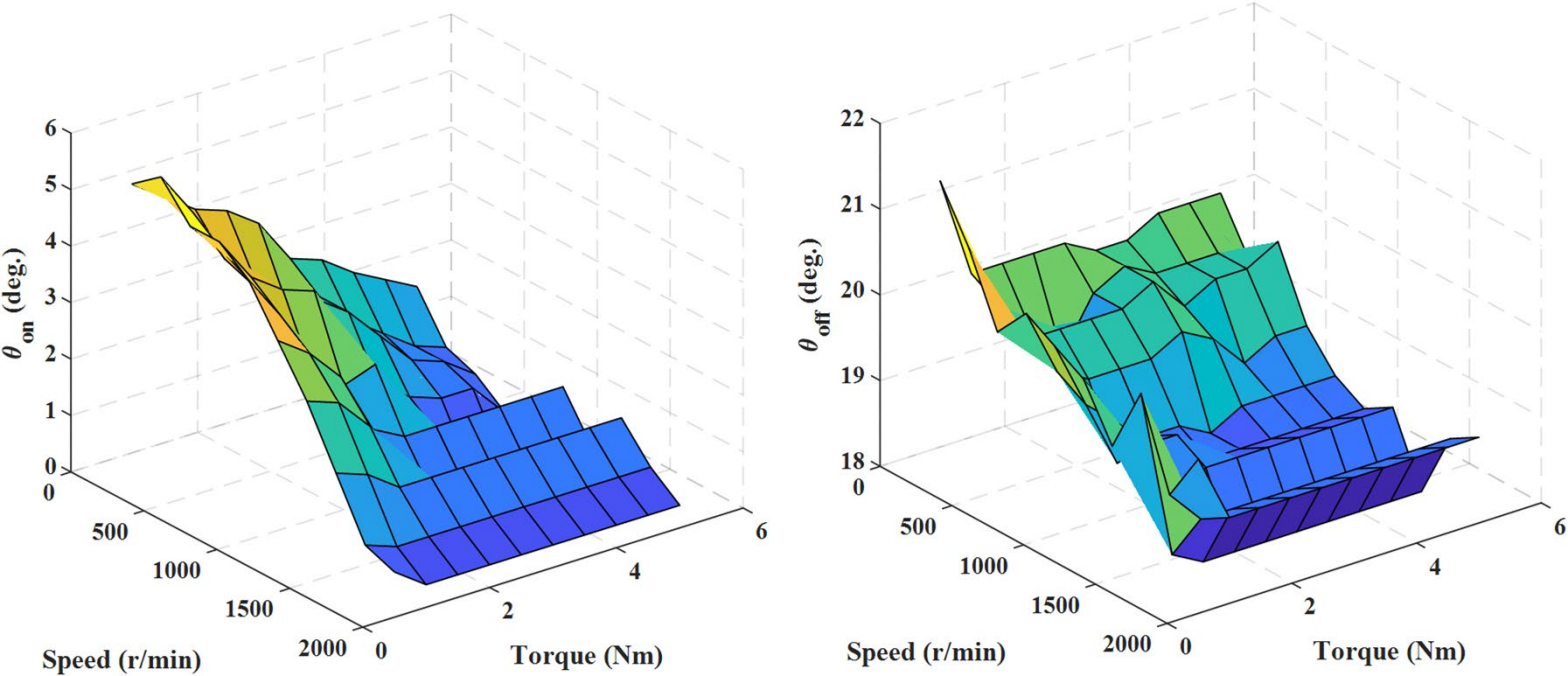
The optimized switching angles.

The optimization process is carried out at each speed (100:100:2000) and reference torque (1:0.5:5 Nm) based on the multi-objective function (7). The population size (N) in the EO algorithm is 25 and the maximum allowed iterations is 35. Subsequently, the optimal switching angles obtained are stored in two look-up tables (LUTs) based on the selected machine’s operating speed and torque ranges, as depicted in Fig. 5.

## Simulation results

The performance of the proposed DITC-WNN is validated through a series of simulations on a three-phase 12/8 SRM prototype; the motor parameters are summarized in Table 1. The simulation results are achieved with a sampling time of 30µs, to match with the experimental results in Sect.  4.

**Table 1:** Parameters of SRM.

Parameter	SRM	Parameter	SRM
Stator/rotor poles	12/8	Stator outer diameter	141 mm
Rated speed	800 r/min	Rotor outer diameter	82 mm
Rated torque	4 Nm	Stator pole width	10 mm
Max phase current	9 A	Rotor pole width	12 mm
Phase resistance	1.73 Ω	Stack length	71 mm

### Case 1: Detailed performance analysis of proposed DITC-WNN

**Fig. 6:**
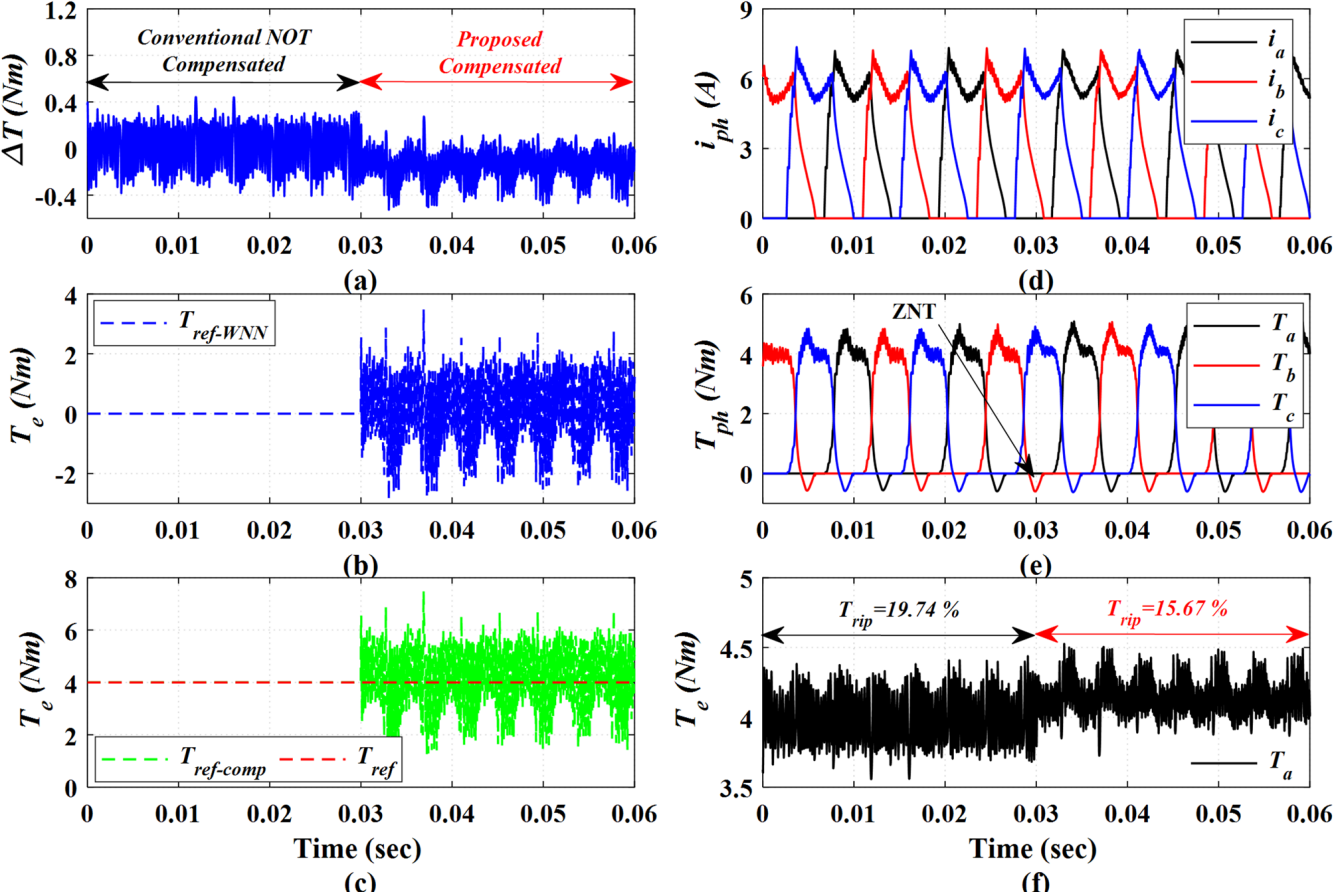
The simulation results explain the performance of Prop-DITC (**a**) torque error, (**b**) output of WNN, (**c**) compensated reference torque, (**d**) phases currents, (**e**) phase torques, (**f**) total generated torque.

Figure 6 describes the detailed operational principles of the proposed DITC-WNN. First, both the proposed DITC-WNN and the conventional DITC approaches are implemented within a parallel coding framework, enabling seamless switching between the two algorithms. The results in the first half of Fig. 6 depict the conventional DITC, where the reference torque is NOT compensated; the results in the second half of Fig. 6 depict the proposed DITC-WNN. Consequently, the motor operates under the conventional DITC for 0.3 s, and then the control is switched to the proposed DITC-WNN approach after a time of 0.3 s., as shown in Fig. 6a. The motor is operating at a speed of 500 r/min and a loading torque of 4 Nm. The profile of torque error in Fig. 6a demonstrates an excellent response of the proposed DITC-WNN, yielding low torque error and the minimum torque ripples. This improvement is attributed to the torque reference compensation incorporated by the proposed WNN.

The torque error is processed by the proposed WNN. After 0.3 s, the WNN outputs the compensated reference torque signal as depicted in Fig. 6b based on the torque error. The total reference torque that is inputted to HTC is also compensated, see Fig. 6c, achieving reduced torque error as presented in Fig. 6a. The online torque compensation based WNN controls HTC and hence controls the current profiles. The current profiles in Fig. 6d may look similar; however, for the proposed DITC-WNN, the current profiles are much smoother, reflecting better current controllability and hence better torque control; this is also approved by the smoother phase torque profiles in Fig. 6e. The improvements in current and torque profiles are a reflection of the improved torque profile in Fig. 6f; the proposed DITC-WNN is showing a superior performance, improving the torque quality and reducing the torque ripple compared to the conventional DITC.

**Fig. 7:**
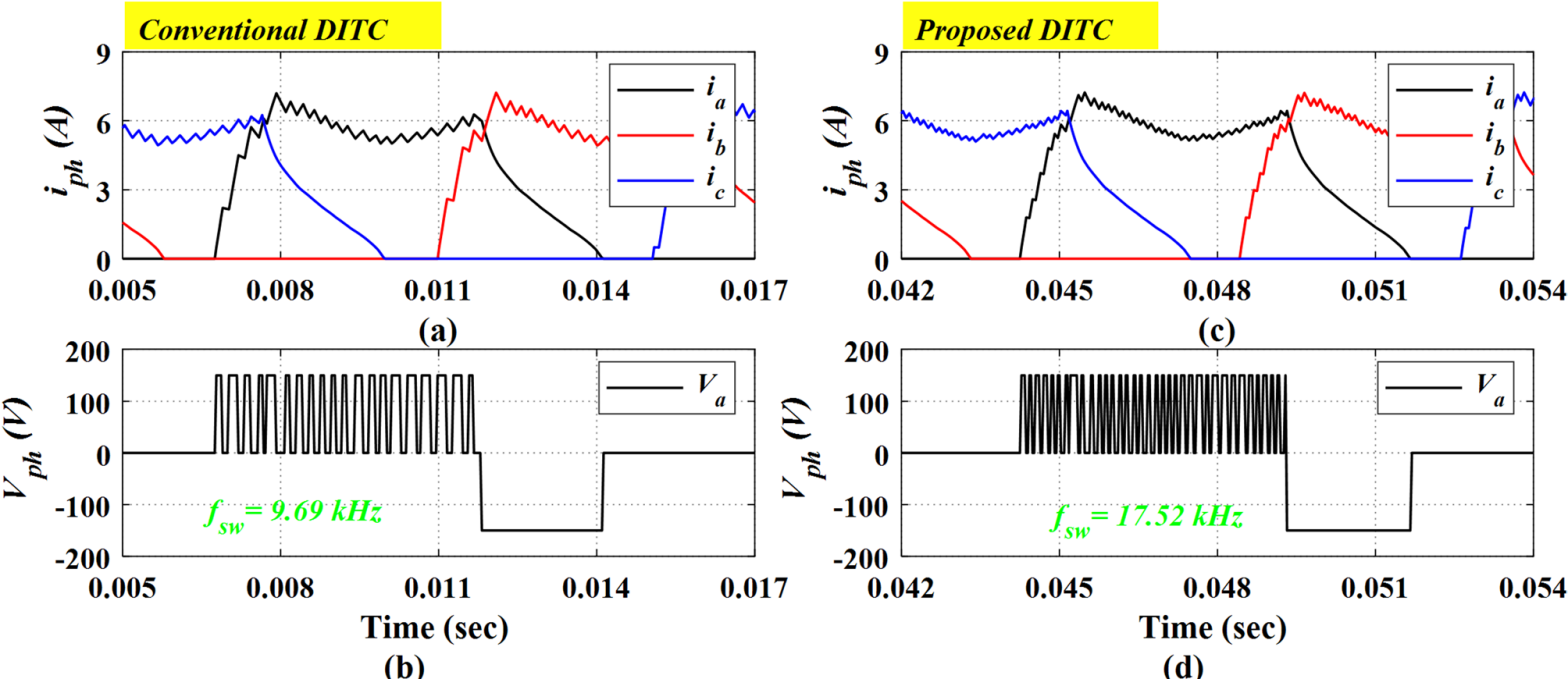
The Simulation results of Prop-DITC and Conv-DITC at 600 r/min (**a**) phase currents for Conv-DITC, (**b**) phase voltage for Conv-DITC, (**c**) phase currents for Prop-DITC, (**d**) phase voltage for Prop-DITC.

Figure 7 investigates the switching frequency ($\:{f}_{sw}$) for Prop-DITC and Conv-DITC schemes by showing the detailed switching states over the current and voltage waveforms. The Conv-DITC exhibits a lower switching frequency ($\:{f}_{sw}$) of 9.69 kHz compared to 17.52 kHz for the proposed DITC. Although the switching frequency in the proposed method is approximately 80% higher, it remains within the acceptable operating range. The higher $\:{f}_{sw}$ for Prop-DITC allows for better current control and hence better torque profiling and improved ripple reduction. The switching frequency of power converter is calculated as:

10 $$\:{f}_{sw}=\frac{1}{\tau\:}{\int\:}_{0}^{\tau\:}{N}_{T}dt$$

where *N*_*T*_ is the total switching number of power converter over one electric period (*τ*).

### Case 2: Performance evaluation at low-speed

**Fig. 8:**
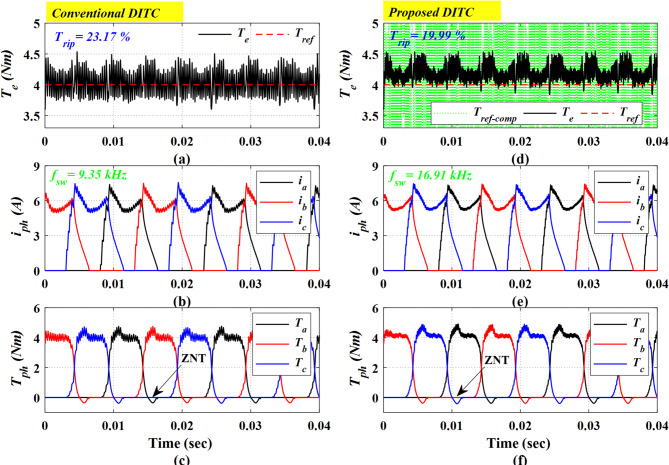
The Simulation results for Prop-DITC and Conv-DITC at speed of 500 r/min and 4 Nm (**a**) torque profile for Conv-DITC (**b**) phase currents for Conv-DITC, (**c**) phase torques for Conv-DITC, (**d**) torque profile for Prop-DITC (**e**) phase currents for Prop-DITC, (**f**) phase torques for Prop-DITC.

Figure 8 shows a comparative simulation result of the torque response, phase currents, and phase torque profile for both the Conv-DITC and Prop-DITC schemes at 500 r/min and 4 Nm. The Prop-DITC exhibits a noticeably smoother torque profile compared to Conv-DITC, as shown in Fig. 8a,d; the Prop-DITC has a torque ripple of 19.99% compared to 23.17% for the Conv-DITC. The torque reduction ratio is calculated as 13.72%. Additionally, the Prop-DITC offers improved phase current and phase torque profiles compared to Conv-DITC. The switching frequencies for both the Conv-DITC and the Prop-DITC schemes are 9.35 kHz and 16.91 kHz (80% higher), respectively.

### Case 3: Performance evaluation at high-speed

**Fig. 9:**
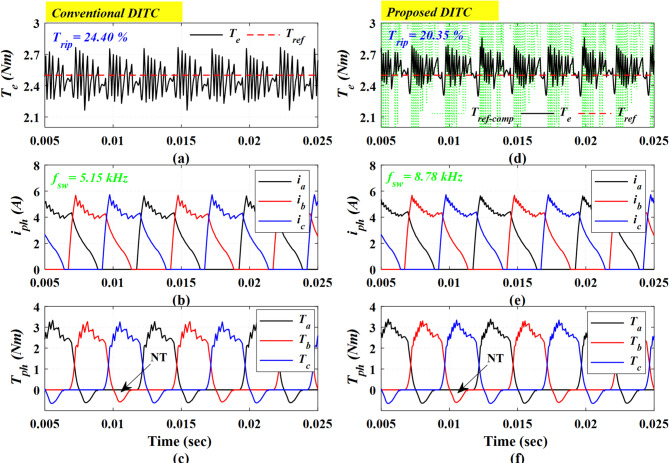
The simulation results comparing the Prop-DITC and Conv-DITC at speed of 1000 r/min and 2.5 Nm (**a**) torque profile for Conv-DITC (**b**) phase currents for Conv-DITC, (**c**) phase torques for Conv-DITC, (**d**) torque profile for Prop-DITC (**e**) phase currents for Prop-DITC, (**f**) phase torques for Prop-DITC.

Figure 9 examines the performance at a higher speed of 1000 r/min and a load of 2.5 Nm. Under these conditions, the Prop-DITC offers a smoother torque profile, with a torque ripple of 20.35% compared to 24.40% for Conv-DITC as shown in Fig. 9a,d. The torque reduction ratio is 16.59%. Besides, the Prop-DITC is showing better profiles for both the phases’ currents and torques. As the speed increases, the switching frequency decreases due to the higher back-emf and the limited current control; the Conv-DITC has $\:{f}_{sw}$ of 5.15 kHz compared to 8.78 kHz (70% higher) for Prop-DITC.

### Case 4: Quantitative analysis based simulation results

**Fig. 10:**
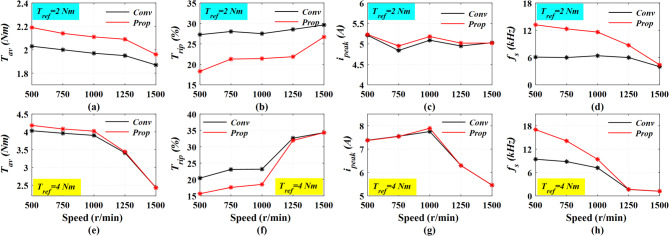
The simulation results comparing the Prop-DITC and Conv-DITC techniques (**a**) average torque with *T*_*ref*_ = 2 Nm, (**b**) torque ripples with *T*_*ref*_ = 2 Nm, (**c**) peak phase current with *T*_*ref*_ = 2 Nm, (**d**) switching frequency with *T*_*ref*_ = 2Nm, (**e**) average torque with *T*_*ref*_ = 4 Nm, (**f**) torque ripples with *T*_*ref*_ = 4 Nm, (**g**) peak phase current with *T*_*ref*_ = 4 Nm, (**h**) switching frequency with *T*_*ref*_ = 4 Nm.

The comparison in Fig. 10 is comparing the proposed and conventional DITC approaches; the simulations are achieved at wide range of operating speeds from 500 r/min to 1500 r/min; also, at different loading torques of 2 Nm and 4 Nm. The comparison involves the average torque, torque ripple, and peak phase current.

First, at loading torque of 2 Nm (*T*_*ref*_ = 2 Nm), the Prop-DITC is providing higher average torque due to torque compensated signal of WNN, see Fig. 10a; it can provide the commanded reference torque (*T*_*ref*_ = 2 Nm) over the complete speed range. On the other side, the Conv-DITC fails to deliver the desired reference torque (*T*_*ref*_ = 2 Nm) at high speeds after 1000 r/min; its provided average torque (*T*_*av*_) is less than *T*_*ref*_. In Fig. 10b, the Prop-DITC is showing a substantial reduction in torque ripples compared to Conv-DITC, reflecting the superior performance of proposed WNN and its proposed training procedure. In Fig. 10c, the peak phase currents are almost identical that is also an advantage for the Prop-DITC, as it reduces the torque ripples without drawing additional currents. Figure 10d demonstrated that the switching frequency of the Prop-DITC approach is considerably higher than the conv-DITC scheme, owing to the WNN offering an online compensation of the reference torque, which results in faster correction of torque error, especially in the commutation region. Consequently, the WNN controller responds more actively to torque error variation, leading to more frequent switching actions than the conventional DITC strategy.

Second, at a heavy loading torque of 4Nm (*T*_*ref*_ = 4Nm), the Prop-DITC still provides higher average torques compared to Conv-DITC as shown in Fig. 10e. Besides, the Prop-DITC reduces the torque ripple significantly, see Fig. 10f. At higher speed of 1500 r/min, the torque ripples are the same for both the Conv-DITC and Prop-DITC due to the uncontrolled phase current, the current control becomes difficult as the speed increases due to the limited dc-link voltage and the increased back-emf. The peak phase currents in Fig. 10g are almost identical. The switching frequency in Prop-DITC is still higher than Conv-DITC, especially at low speed, as shown in Fig. 10h.

## Experimental validations

**Fig. 11:**
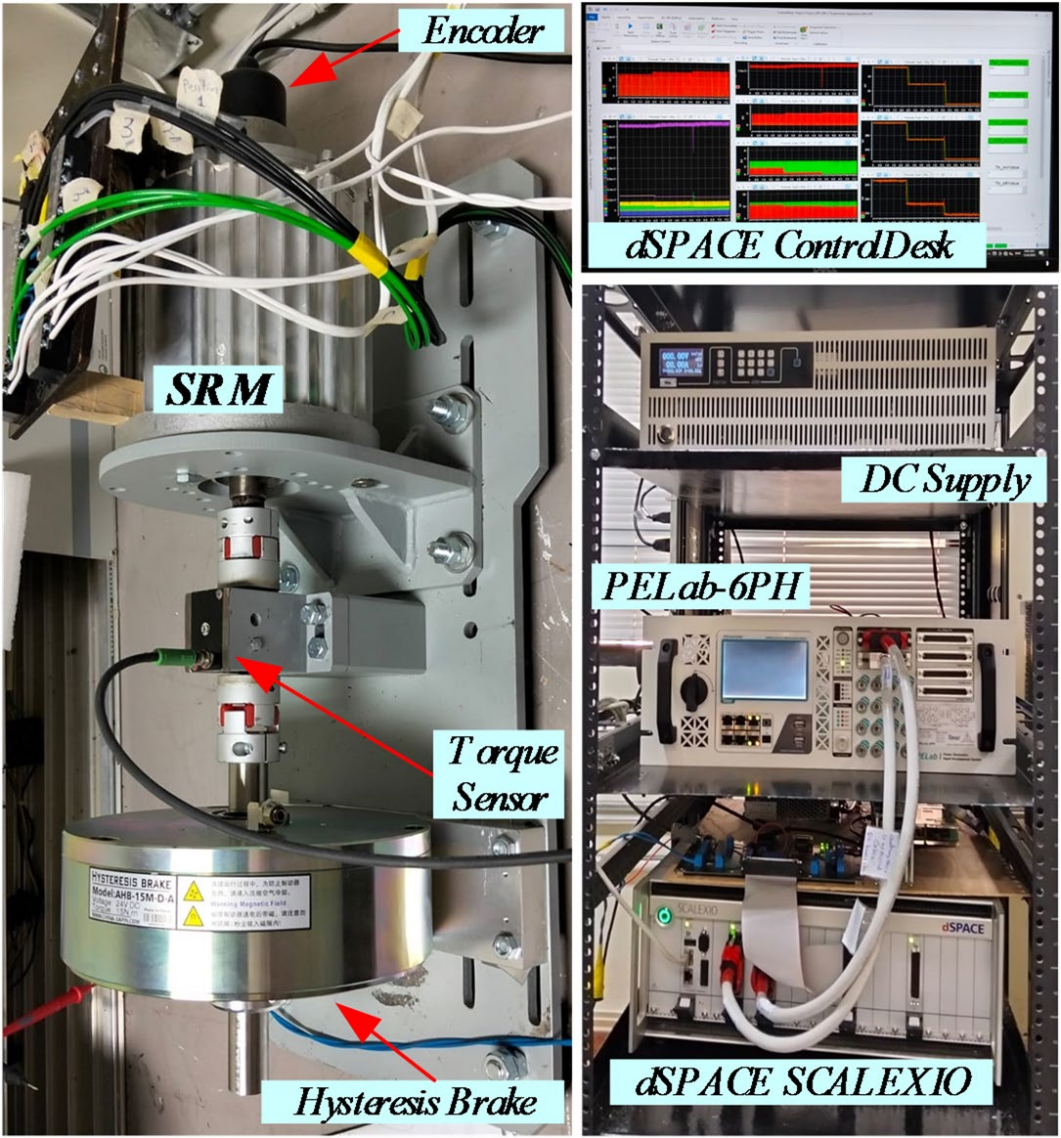
The experimental testbed.

The experimental testbed, illustrated in Fig. 11, comprises a three-phase 12/8 SRM prototype, a torque transducer, a hysteresis brake, a 3600-PPR incremental encoder, a DC power supply, and a SCALEXIO dSPACE control unit. The setup also includes current and voltage sensors, along with a six-phase PELAB-6PH inverter that is configured to drive the SRM. Real-time data acquisition and monitoring are performed using dSPACE ControlDesk, then plotted using MATLAB.

The experimental verifications were conducted to ensure a fair comparison between the Conv-DITC and Prop-DITC schemes; a unified testing framework is designed by implementing the two control algorithms (Conv-DITC and Prop-DITC) in parallel form within a single real-time environment, then a switching mechanism is employed to transit between them; ensuring that the motor was subjected to the identical dynamic and steady-state conditions in both scenarios.

### Case 1: Performance evaluation under open loop torque control

In the open loop torque control, the reference torque (*T*_*ref*_) is the direct input signal, the outer loop speed controllers in Figs. 1 and 2 are not involved here. The motor speed is adjusted by controlling the loading torque by the hysteresis brake unit. The reason for considering the open loop torque control is that it provides a pure reference torque signal, hence providing clearer observations and considerations for the torque ripples.

**Fig. 12:**
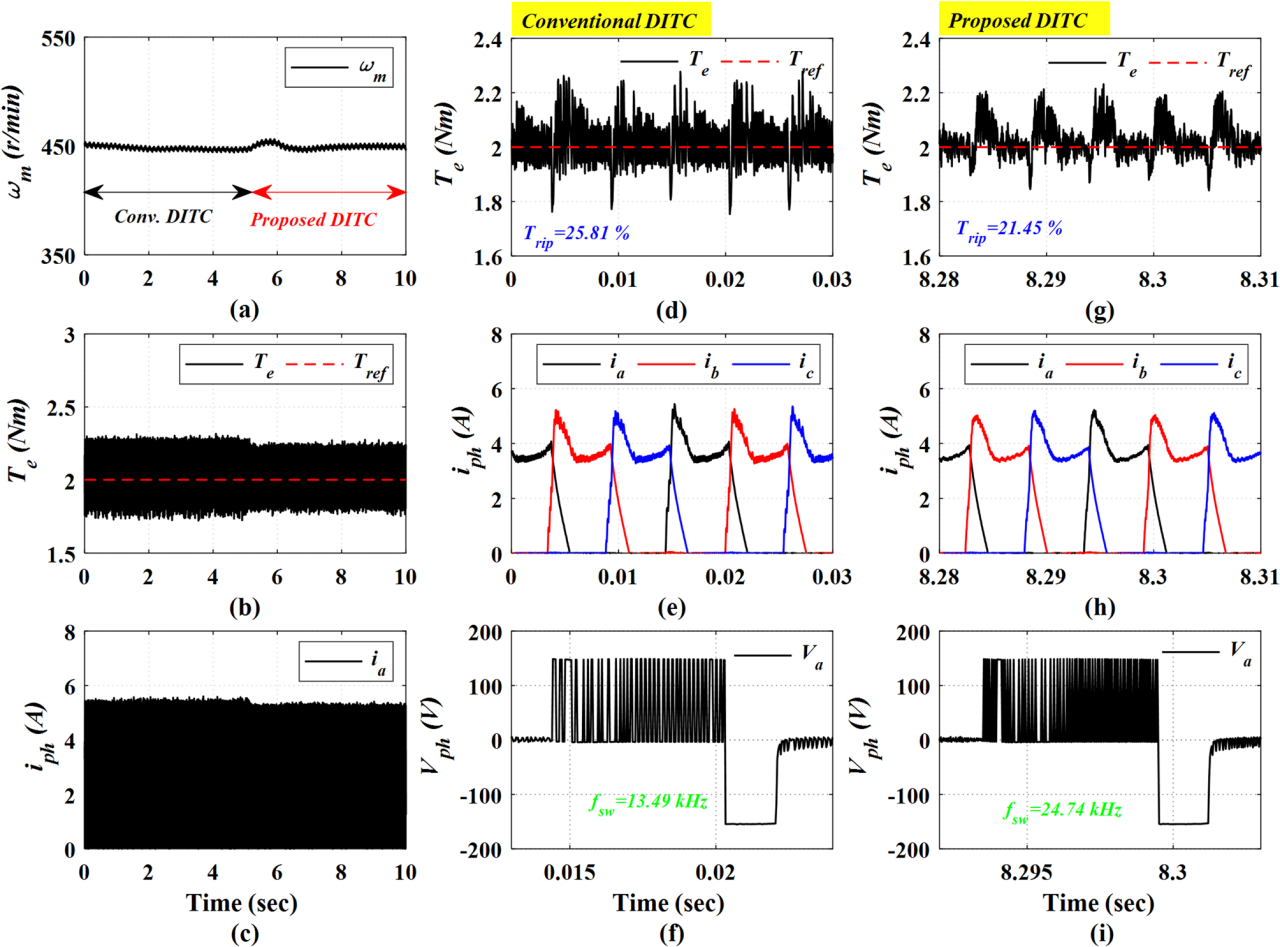
The Experimental Results under open loop torque control at speed of 450 r/min and 2 Nm, (**a**) Speed, (**b**) Torque, (**c**) phase current, (**d**) Torque profile for conv-DITC, (**e**) Phase current profile for conv-DITC, (**f**) Phase voltage profile for conv-DITC, (**g**) Torque profile for Prop-DITC, (**h**) Phase current profile for Prop-DITC, (**i**) Phase voltage profile for Prop-DITC.

In Fig. 12, under open-loop torque control, the motor is running at a speed of 450 r/min and a loading torque of 2 Nm. A transition between the Conv-DITC and Prop-DITC is achieved at a time of 5 s; first, the motor is running considering the Conv-DITC algorithm from 0 s till time of 5 s, then the Prop-DITC is considered from 5 s till 10 s, see Fig. 12b. The motor speed is nearly constant through the test, showing only minor variations due to the absence of a closed-loop speed control, see Fig. 12a. In Fig. 12b, the Prop-DITC indicates a clear enhancement in torque profile. The peak currents are almost the same as depicted by Fig. 12c. The torque ripple of Conv-DITC is 25.81%, as depicted in Fig. 12d, compared to 21.45% for Prop-DITC, as presents in Fig. 12g, corresponding to a ripple-reduction ratio of 16.89%. This reduction demonstrates the capability of the Prop-DITC approach in providing a smoother torque profile under open-loop conditions. The Prop-DITC yields a smoother current profile as shown in Fig. 12h compared to that of Conv-DITC in Fig. 12e. Figure 12f, i illustrate the phase voltages for the Conv-DITC and Prop-DITC schemes, respectively. These figures also depict the corresponding switching states for each control strategy, thereby enabling an assessment of their respective switching frequencies. The Conv-DITC exhibits a switching frequency of 13.49 kHz, whereas the Prop-DITC shows a switching frequency of 24.74 kHz, which is approximately 83% higher. This observation is also in close agreement with the simulation results presented in Fig. 7.

**Fig. 13:**
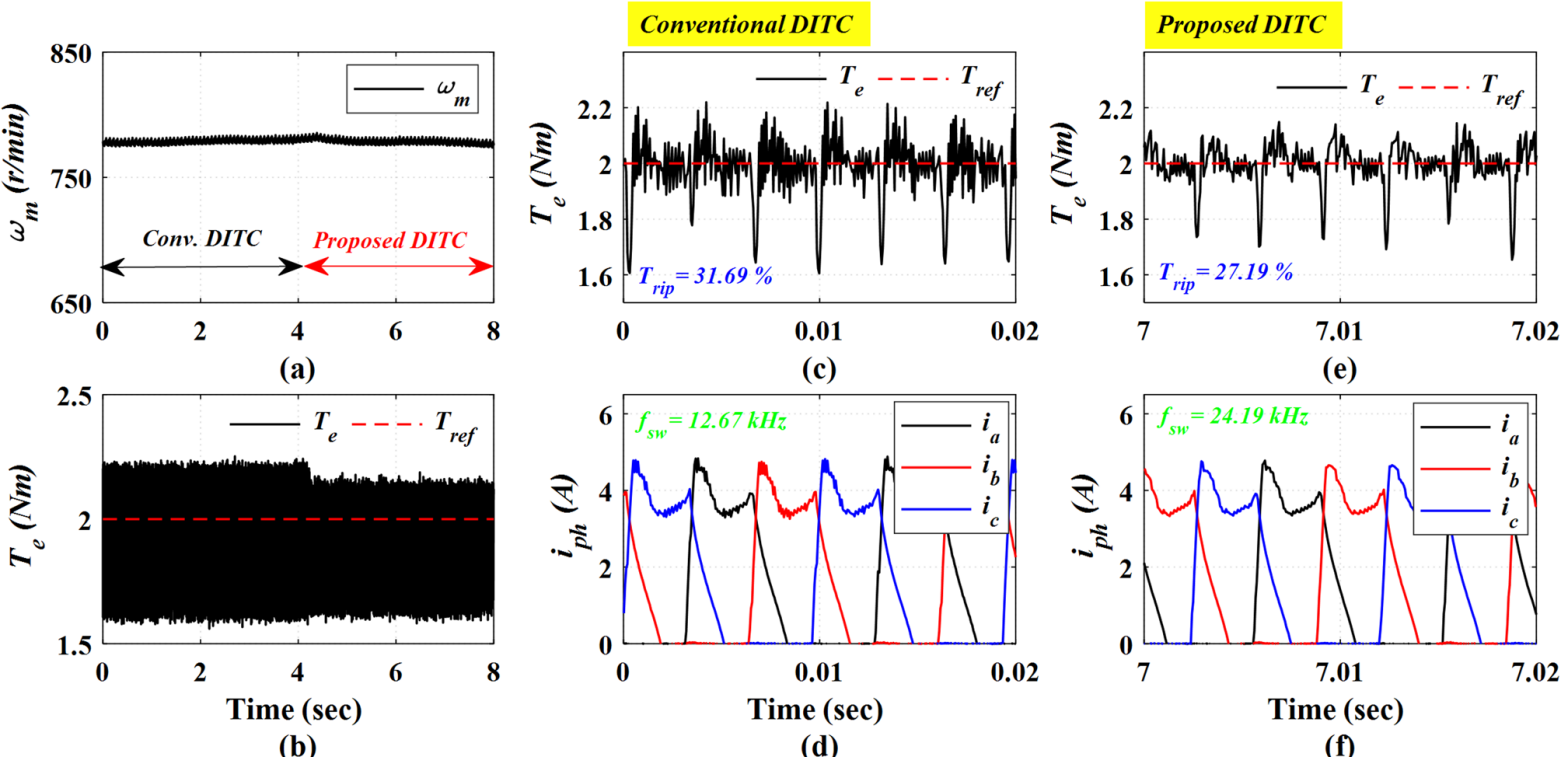
The experimental results under open loop torque control at speed of 780 r/min and 2 Nm, (**a**) Speed, (**b**) Torque, (**c**) Torque profile for conv-DITC, (**d**) Phase current profile for conv-DITC, (**e**) Torque profile for Prop-DITC, (**f**) Phase current profile for Prop**-**DITC.

Figure 13 demonstrates the experimental results at a speed of 780 r/min (rated speed is 800 r/min) and a load torque of 2 Nm. The Prop-DITC still presents a notable improvement in torque profile, as shown in Fig. 13b, with a lower torque ripple of 27.19% compared to 31.69% for Conv-DITC, as shown in Fig. 13c,e. The minimization ratio of torque ripples is 14.20% due to the compensated torque reference based on the WNN controller.

**Fig. 14:**
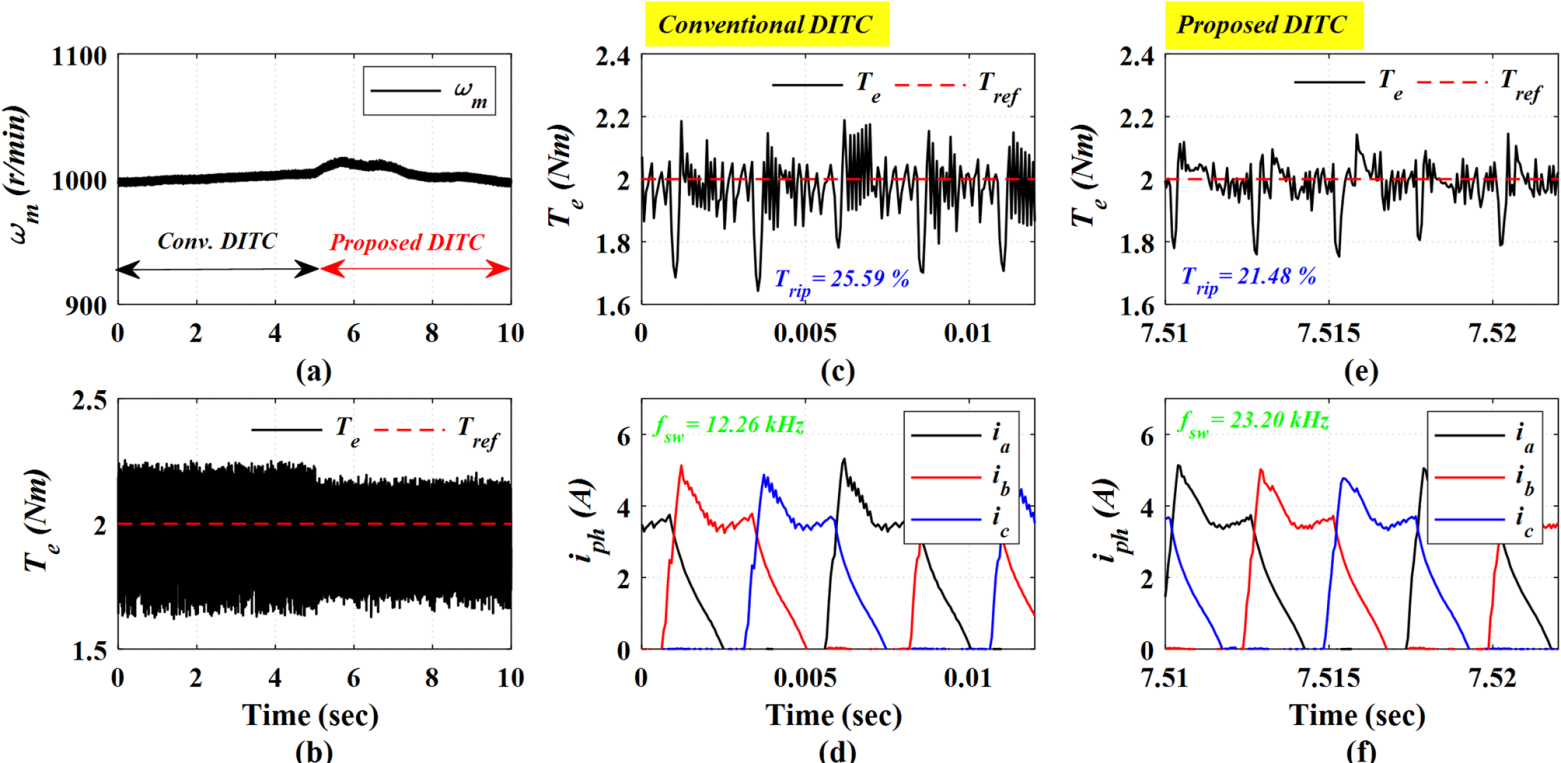
The experimental results under open loop torque control at speed of 1000 r/min and 2 Nm, (**a**) Speed, (**b**) Torque, (**c**) Torque profile for conv-DITC, (**d**) Phase current profile for conv-DITC, (**e**) Torque profile for Prop-DITC, (**f**) Phase current profile for Prop-DITC.

Figure 14 presents the experimental results at a high speed of 1000 r/min and a load torque of 2 Nm. The Prop-DITC demonstrates a remarkable enhancement in torque quality as illustrated in Fig. 14b, yielding a reduced torque ripple of 21.48% compared to 25.59% for Conv-DITC, as shown in Fig. 14c,e. The reduction ratio of torque ripples is 16.06%.

**Fig. 15:**
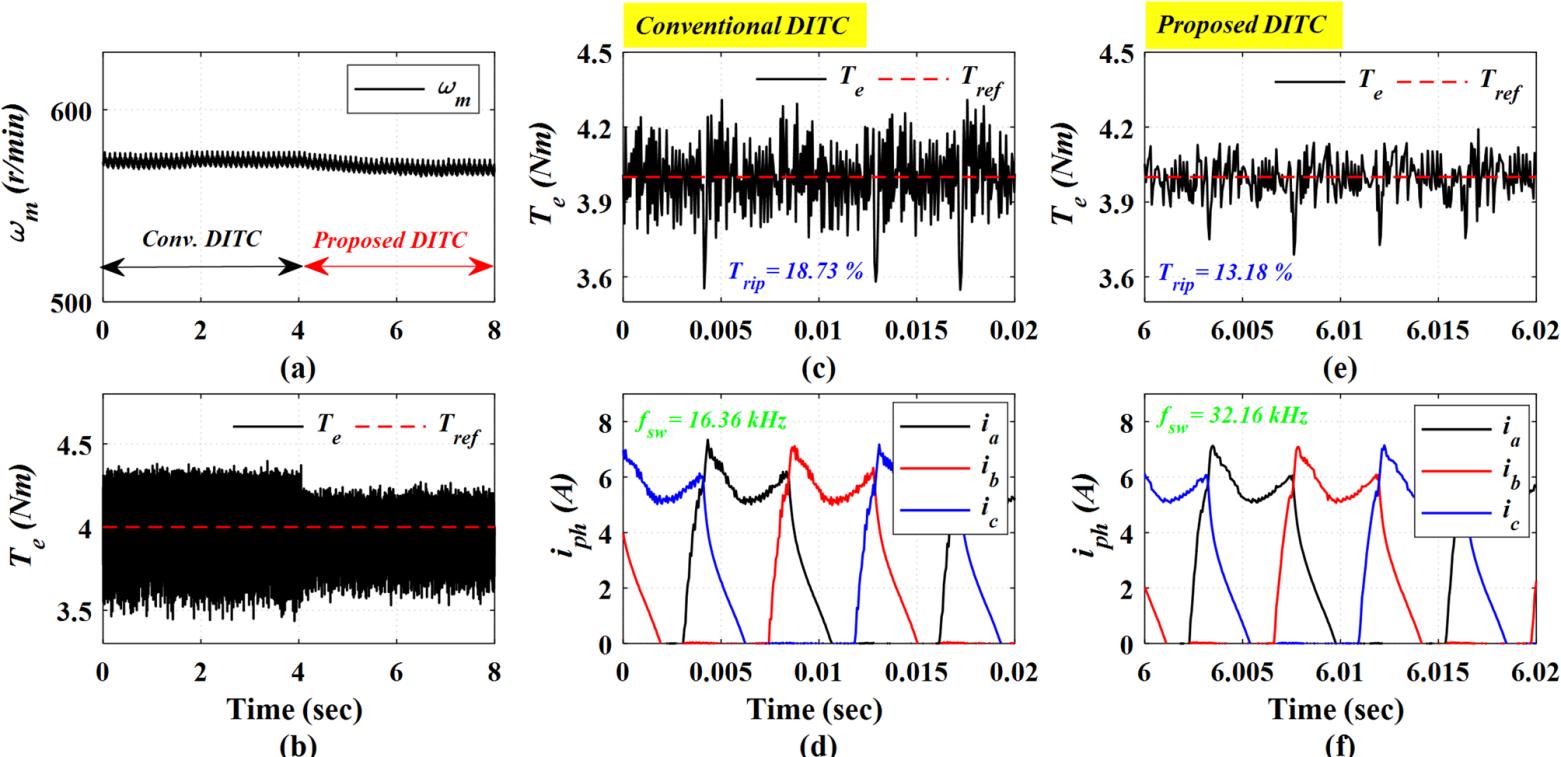
The experimental results under open loop torque control at speed of 575 r/min and 4 Nm, (**a**) Speed, (**b**) Torque, (**c**) Torque profile for conv-DITC, (**d**) Phase current profile for conv-DITC, (**e**) Torque profile for Prop-DITC, (**f**) Phase current profile for Prop-DITC.

Figure 15 presents the experimental results at a low speed of 575 r/min and a heavy load torque of 4 Nm. The Prop-DITC continues to exhibit a substantial improvement in torque quality as depicted in Fig. 15b, achieving a torque ripple of 13.18% compared to 18.73% with the Conv-DITC, as shown in Fig. 15c,e; this corresponds to a torque-ripple reduction of 29.63%, further demonstrating the ability of the Prop-DITC approach under high-load operating conditions.

**Fig. 16:**
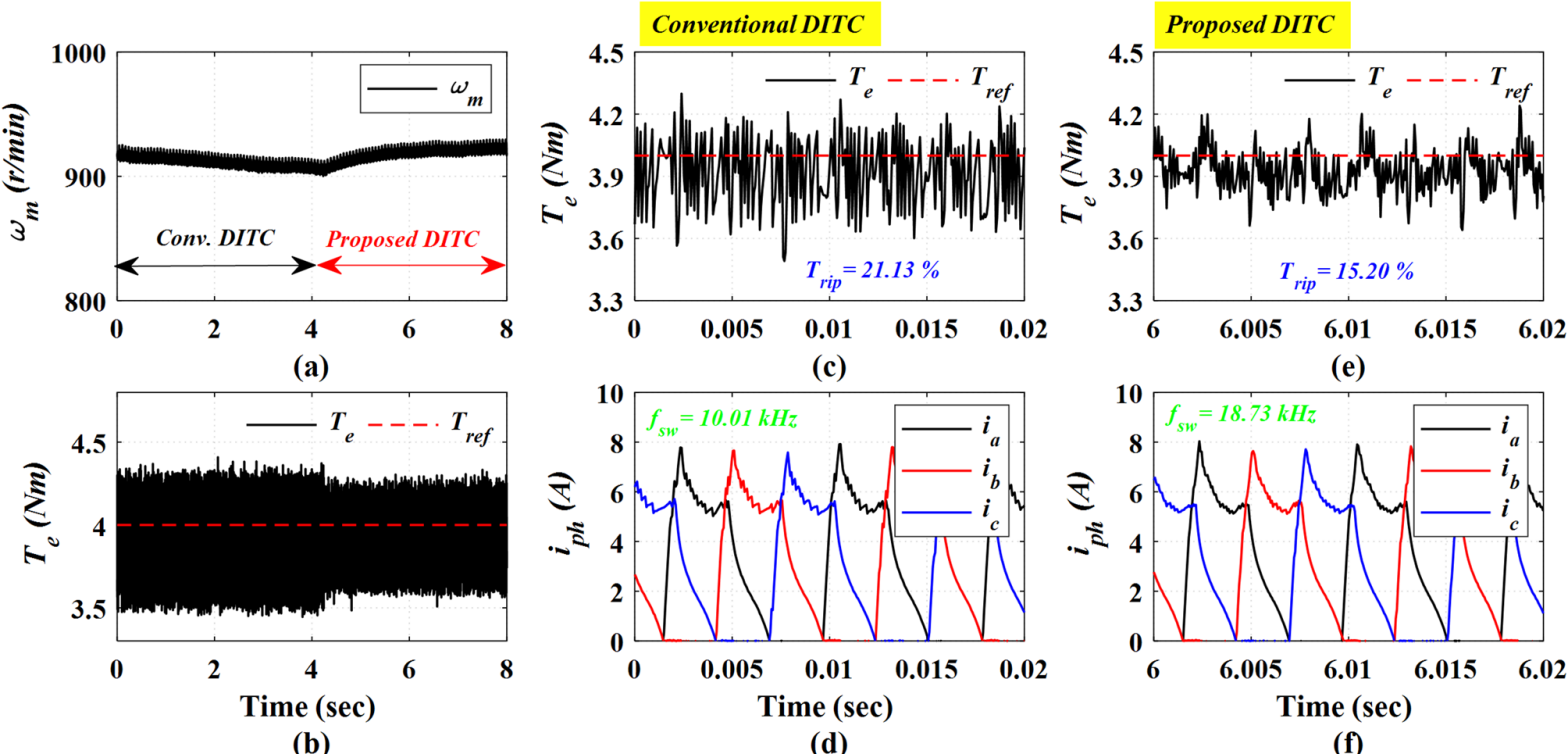
The experimental results under open loop torque control at speed of 920 r/min and 4 Nm, (**a**) Speed, (**b**) Torque, (**c**) Torque profile for conv-DITC, (**d**) Phase current profile for conv-DITC, (**e**) Torque profile for Prop-DITC, (**f**) Phase current profile for Prop-DITC.

Figure 16 depicts the experimental results acquired at a high speed of 920 r/min and a high loading torque of 4 Nm. The Prop-DITC exhibits a substantial improvement in torque quality, achieving a torque ripple of 15.2% compared to 21.13% for the Conv-DITC, as presented in Fig. 16c,e; corresponding to a torque-ripple reduction of 28.06%.

The experimental results under open loop torque control are summarized in Table 2. The Prop-DITC consistently provided better torque profiles with a significant reduction in torque ripples compared to Conv-DITC. This superior performance of Prop-DITC is attributed to the compensated torque signal of proposed WNN. On the other hand, the Prop-DITC exhibits higher switching frequencies than the Conv-DITC, particularly at low speeds, with an increase of approximately 80%. Nevertheless, the switching frequency remains within the acceptable range for industrial applications. Noting that the maximum switching frequency is equal to the sampling frequency of SCALEXIO unit (33.33 kHz).

**Table 2:** Comparative experimental analysis for the Conv-DITC and Prop-DITC under open-loop torque control.

Operating point	*T*_rip_ (%) Conv-DITC	*T*_rip_ (%) Prop-DITC	Reduction ratio of T_rip_ for Prop-DITC		*f*_s_ (kHz) Conv-DITC	*f*_s_ (kHz) Prop-DITC
450 r/min and 2Nm	25.81	21.45	16.89% ↓	Average of 16% for a light load of 2Nm	13.49	24.74
780 r/min and 2Nm	31.69	27.19	14.20% ↓		12.67	24.19
1000 r/min and 2Nm	25.59	21.48	16.06% ↓		12.26	23.20
575 r/min and 4Nm	18.73	13.18	29.63% ↓	Average of 28.8% for heavy load 4Nm	16.36	32.16
920 r/min and 4Nm	21.13	15.2	28.06% ↓		10.01	18.73

### Case 2: Performance evaluation under closed loop control

In this section, the speed controller is activated to present the actual real-time operating conditions. The experimental results are achieved considering different speeds (500 to 1200 r/min) and different loading scenarios (2 to 4 Nm).

**Fig. 17:**
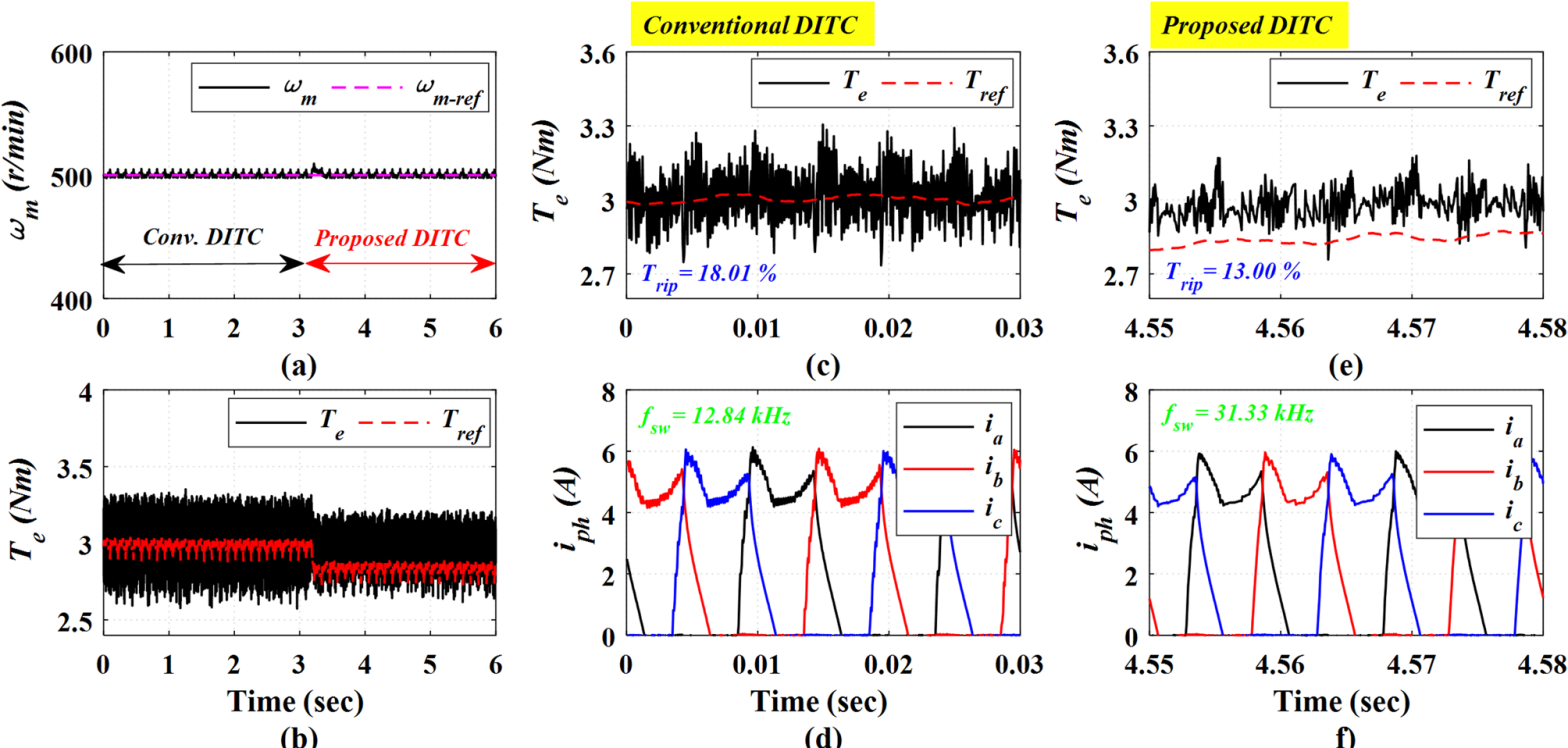
The experimental results under closed loop control at speed of 500 r/min and 3 Nm, (**a**) Speed, (**b**) Torque, (**c**) Torque profile for conv-DITC, (**d**) Phase current profile for conv-DITC, (**e**) Torque profile for Prop-DITC, (**f**) Phase current profile for Prop-DITC.

In Fig. 17, the motor is tested under a low speed of 500 r/min and a loading torque of 3Nm, see Fig. 17a. As depicted in Fig. 17b, the Prop-DITC approach offers a remarkably smoother torque profile compared to the Conv-DITC method. The Conv-DITC torque profile, demonstrated in Fig. 17c, presents a torque ripple of 18.01%, while the Prop-DITC produces a reduced torque ripple of 13%, as shown in Fig. 17e; The torque-ripple mitigation ratio of 27.81% exhibits the capability of the WNN-DITC scheme in achieving a smoother torque profile under a closed-loop operation. Whereas the corresponds phase current profiles, depicted in Fig. 17d, f, further approve that the proposed DITC strategy delivers a notably smoother current waveform.

**Fig. 18:**
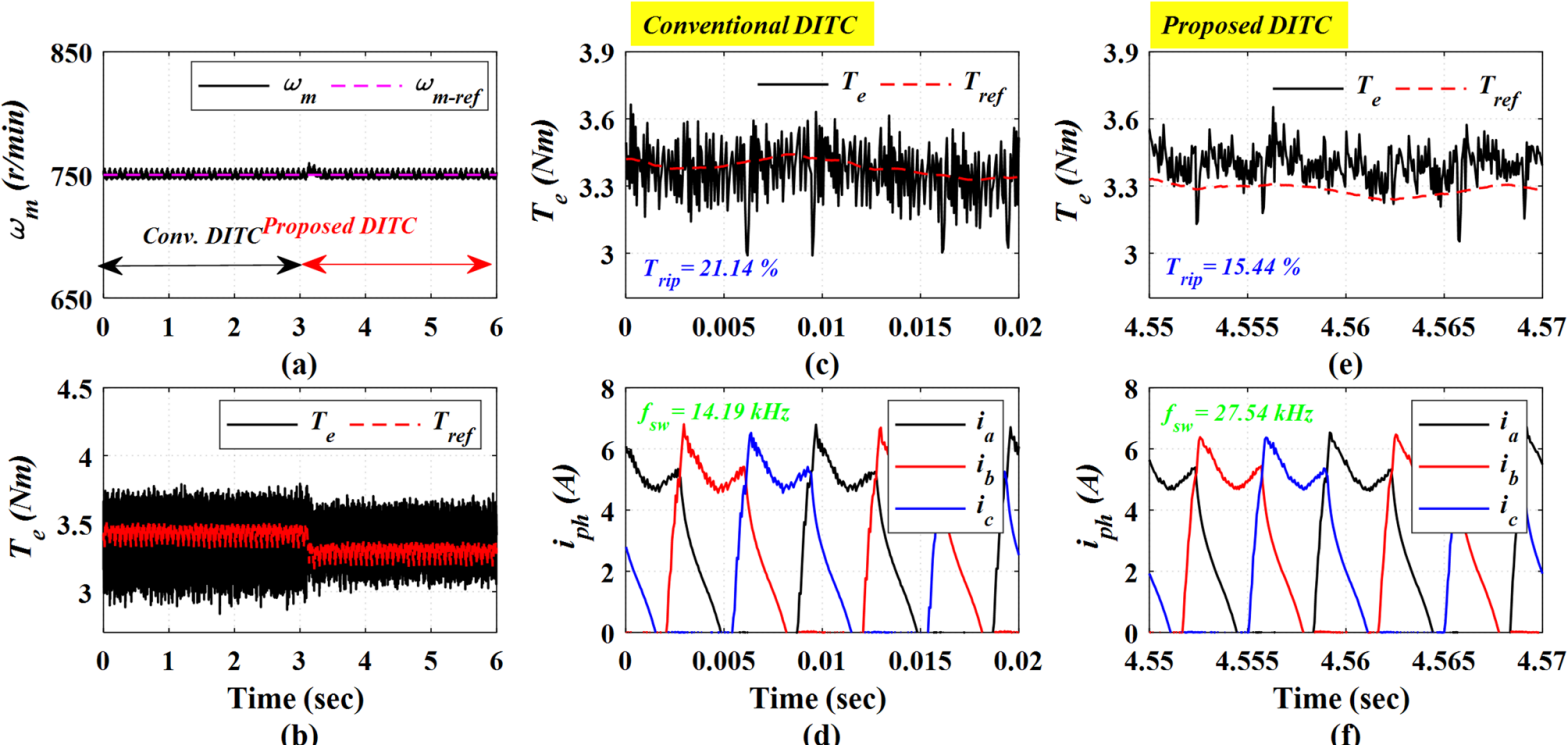
The experimental results under closed loop control at speed of 750 r/min and 3.5 Nm, (**a**) Speed, (**b**) Torque, (**c**) Torque profile for conv-DITC, (**d**) Phase current profile for conv-DITC, (**e**) Torque profile for Prop-DITC, (**f**) Phase current profile for Prop**-**DITC.

Figure 18 presents the experimental results obtained at a medium speed of 750 r/min under a heavy load torque of 3.5 Nm. The Prop-DITC demonstrates a superior torque profile with significant reduced torque ripple. As presented in Fig. 18c,e, the torque ripple minimizes from 21.14% with the Conv-DITC to 15.44% with the Prop-DITC, corresponding to a reduction ratio of 26.96%.

**Fig. 19:**
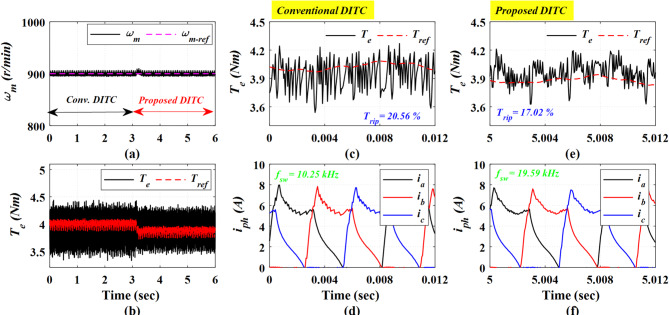
The experimental results under closed loop control at speed of 900 r/min and 4 Nm, (**a**) Speed, (**b**) Torque, (**c**) Torque profile for conv-DITC, (**d**) Phase current profile for conv-DITC, (**e**) Torque profile for Prop-DITC, (**f**) Phase current profile for Prop-DITC.

The experimental results at high speed of 900 r/min and a heavy loading torque of 4 Nm are shown in Fig. 19. The Prop-DITC still shows a superior ability to reduce torque ripples with a 17.02% compared to 20.56% for the Conv-DITC, as shown in Fig. 19b,c,e; corresponding to a 17.21% reduction ratio in torque ripples, thus substantiating the capability of Prop-DITC under heavy-loads and high-speed conditions.

**Fig. 20:**
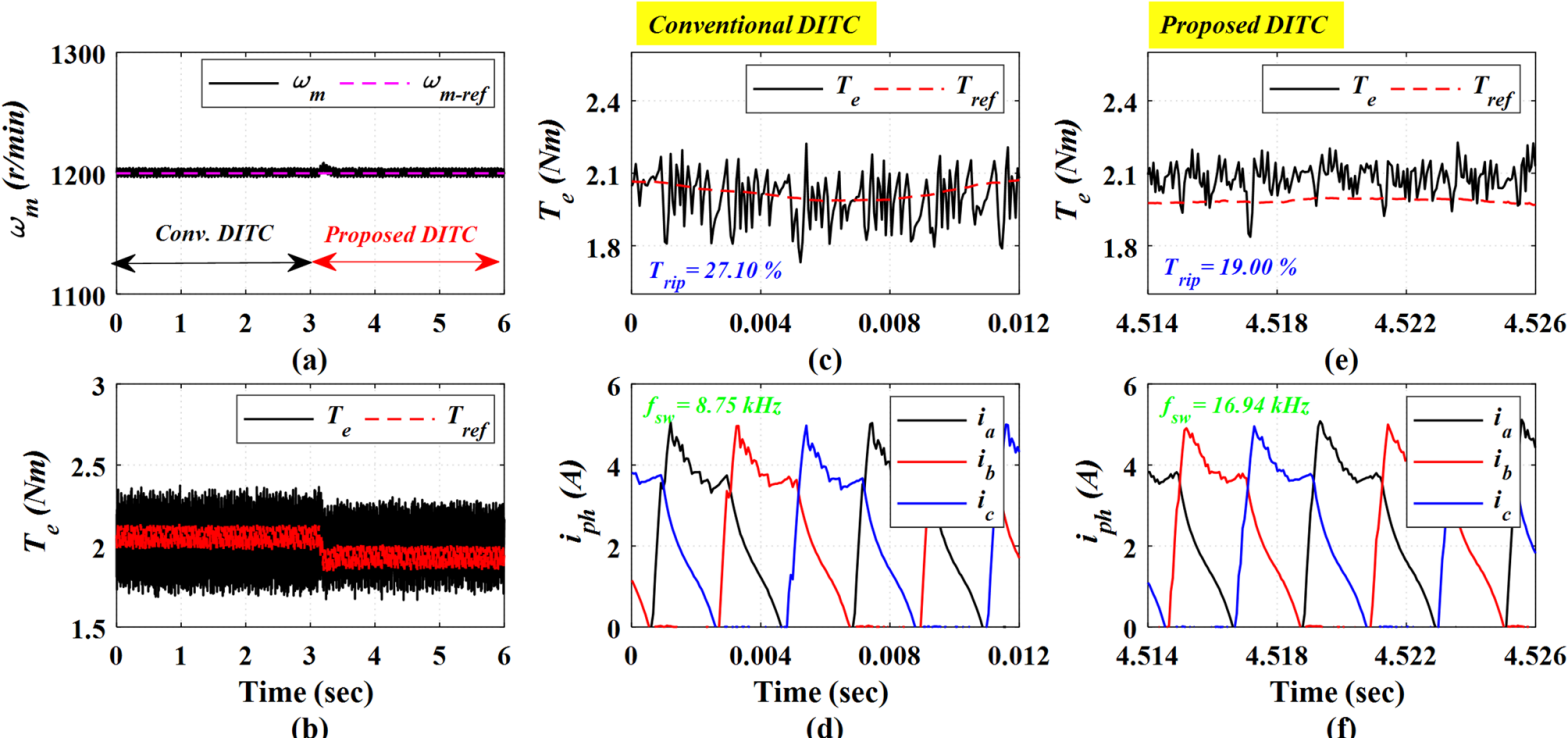
The experimental results under closed loop control at speed of 1200 r/min and 2 Nm, (**a**) Speed, (**b**) Torque, (**c**) Torque profile for conv-DITC, (**d**) Phase current profile for conv-DITC, (**e**) Torque profile for Prop-DITC, (**f**) Phase current profile for Prop-DITC.

Figure 20 presents the experimental results at a high speed of 1200 r/min and a light loading torque of 2 Nm. The Prop-DITC has the ability to provide a better torque profile compared to the Conv-DITC, as depicted in Fig. 20b, exhibiting substantially reduced torque ripple. The Conv-DITC produces a torque ripple of 27.1% (Fig. 20c), whereas the Prop-DITC achieves a reduced ripple ratio of 19% (Fig. 20e). The reduction ratio of torque ripples is 29.88% attributable to the nonlinear compensation capability of the WNN-based reference controller.

**Fig. 21:**
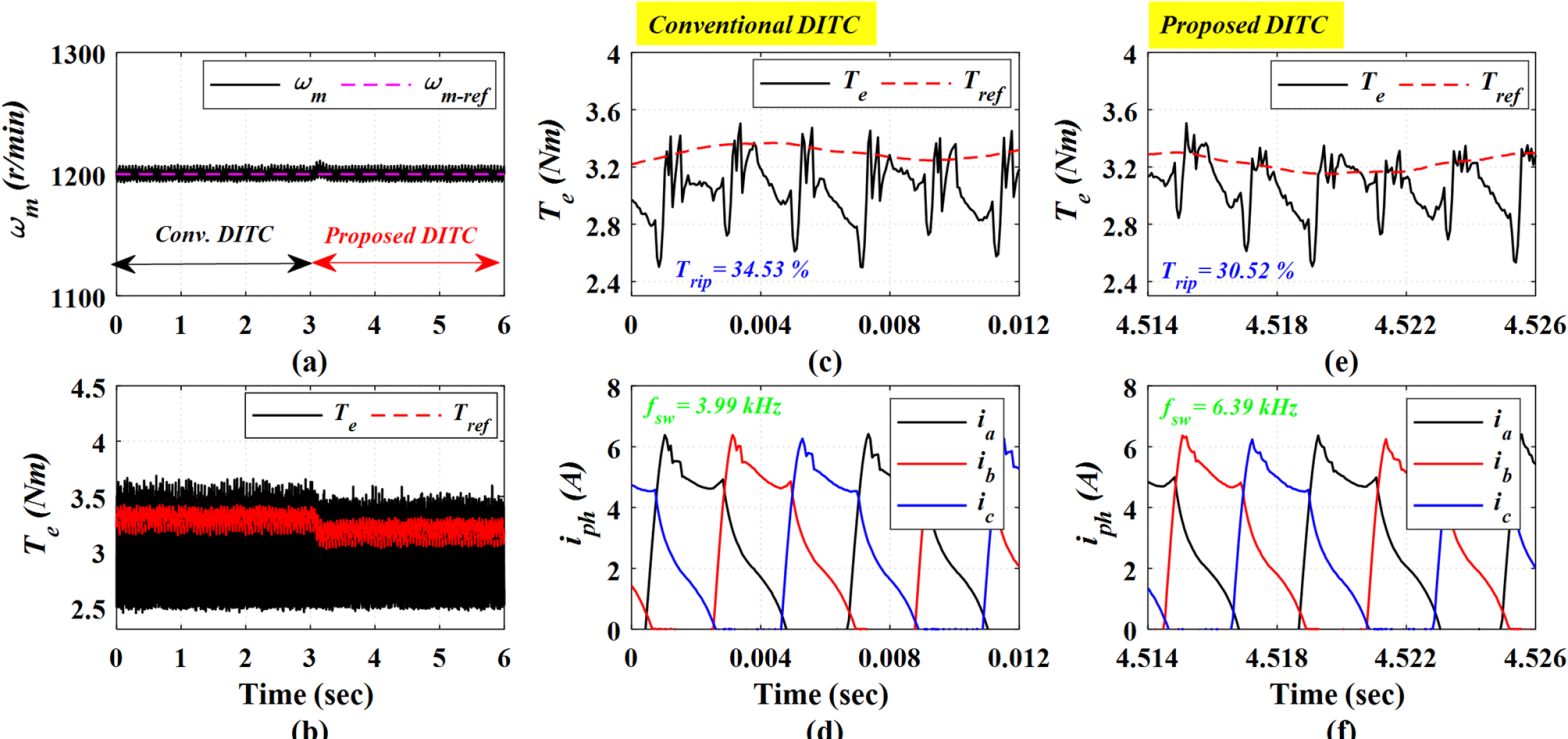
The experimental results under closed loop control at speed of 1200 r/min and 3.2 Nm, (**a**) Speed, (**b**) Torque, (**c**) Torque profile for conv-DITC, (**d**) Phase current profile for conv-DITC, (**e**) Torque profile for Prop-DITC, (**f**) Phase current profile for Prop-DITC.

Figure 21 presents the experimental results obtained at a high speed of 1200 r/min under a heavy load torque of 3.2 Nm. The Conv-DITC yields a torque ripple of 34.53% (Fig. 21c), whereas the Prop-DITC reduces the ripple to 30.52% (Fig. 21e). The reduction ratio of torque ripples is 11.61%, further demonstrating the effectiveness of the proposed WNN-DITC approach under heavy-speed, high-load operating conditions, as depicted in Table 3.

Table 3 summarizes the closed-loop experimental results. The Prop-DITC achieves improved torque characteristics with significantly reduced torque ripple compared with the Conv-DITC, mainly due to the WNN-based torque compensation. Although the Prop-DITC results in a higher switching frequency (240% higher at low speed of 500 r/min, 94% higher at speed of 750 r/min, 90% higher at speed of 900 r/min); however, it remains within the acceptable range of several industrial applications.

**Table 3:** Comparison based on the experimental results for the Conv-DITC and Prop-DITC under closed loop control.

Operating point	*T*_rip_ (%) Conv-DITC	*T*_rip_ (%) Prop-DITC	Reduction ratio of T_rip_ for Prop-DITC	*f*_s_ (kHz) Conv-DITC	*f*_s_ (kHz) Prop-DITC
500 r/min and 3 Nm	18.01%	13.00%	27.81% ↓	12.84	31.33
750 r/min and 3.5 Nm	21.14%	15.44%	26.96% ↓	14.19	27.54
900 r/min and 4 Nm	20.56%	17.02%	17.21% ↓	10.25	19.59
1200 r/min and 2 Nm	27.10%	19.00%	29.88% ↓	8.75	16.94
1200 r/min and 3.2 Nm	34.53%	30.52%	11.61% ↓	3.99	6.39

### Case 3: Closed loop control with step change in reference speed

**Fig. 22:**
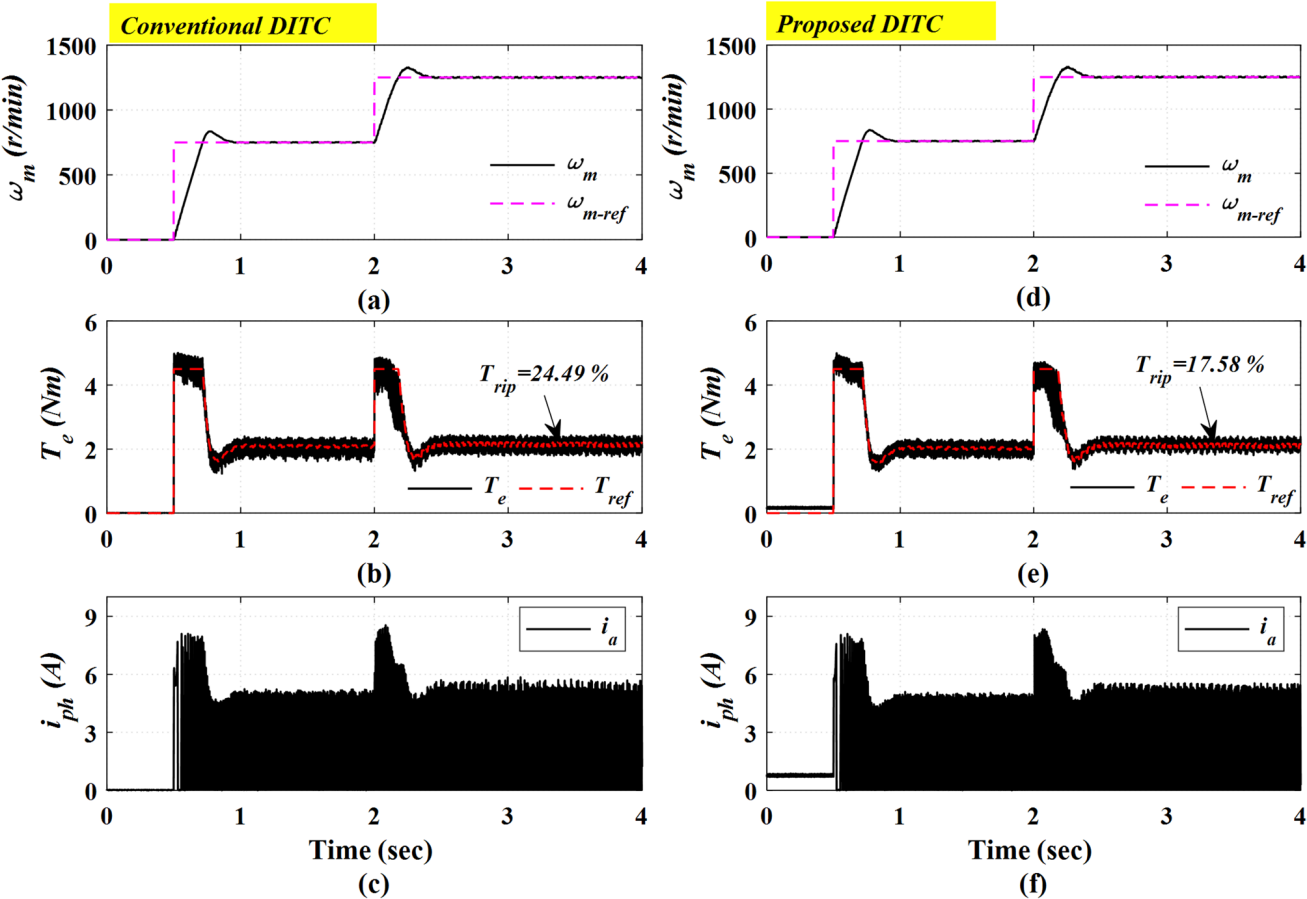
The experimental results with step change in reference speed, (**a**) Speed for conv-DITC, (**b**) Torque for conv-DITC, (**c**) Current for conv-DITC, (**d**) Speed for conv-DITC, (**e**) Torque for Prop -DITC, (**f**) Current for Prop -DITC.

Figure 22 demonstrates the experimental results for a sudden step change in the reference speed. The motor speed is increased from 750 r/min to 1250 r/min at a time of 2 s. while retaining a constant load torque of 2 Nm. The motor starts its operation from standstill, as shown in Fig. 22a for the Conv-DITC and Fig. 22d for the Prop-DITC. A fast dynamic response is observed in the torque profiles, as demonstrated in Fig. 22b,e. Moreover, the Prop-DITC realizes a better torque profile for both low and high-speed operating conditions, maintaining effective torque control tracking and a lower torque ripple of 17.58%, compared with 24.49% for the Conv-DITC.

### Case 4: Closed loop control with sudden change in loading torque

**Fig. 23:**
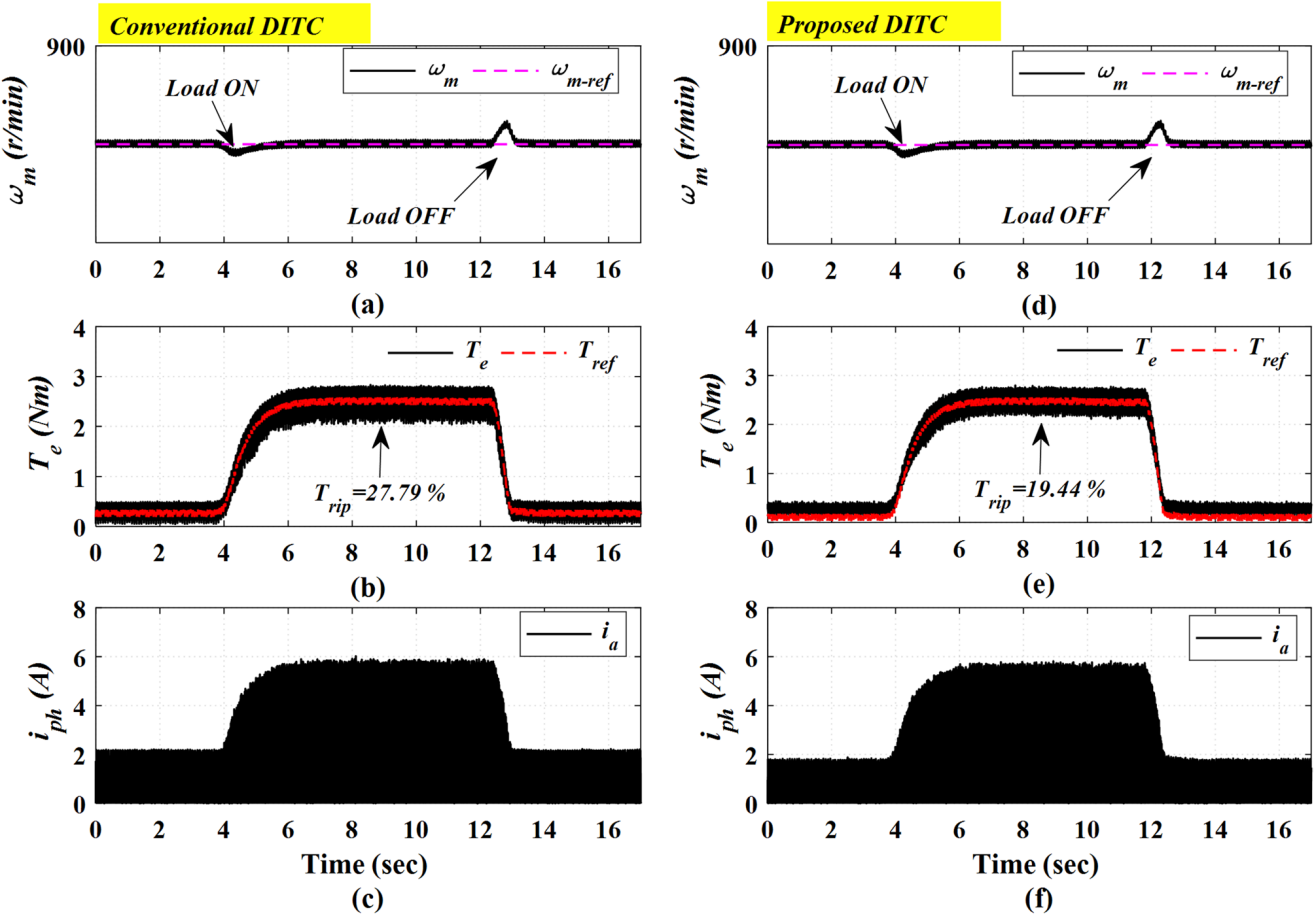
The experimental results with sudden change in loading torque at 750 r/min, (**a**) Speed for conv-DITC, (**b**) Torque for conv-DITC, (**c**) Current for conv-DITC, (**d**) Speed for conv-DITC, (**e**) Torque for Prop-DITC, (**f**) Current for Prop-DITC.

Figure 23 describes the comparative experimental results under a sudden torque loading change. The motor is running at a constant speed of 800 r/min, see Fig. 23a, d, initially under no-load conditions. A loading torque of 3 Nm is applied at 4 s and subsequently removed at 12 s. The Prop-DITC exhibits a faster dynamic response and a smoother torque profile, as illustrated in Fig. 23e, compared with the Conv-DITC shown in Fig. 23b. Furthermore, the Prop-DITC achieves a lower torque ripple of 19.44%, relative to 27.79% for the Conv-DITC, corresponding to a torque-ripple reduction of 30.04%.

It is worth mentioning that the real-time feasibility of the proposed WNN-DITC framework was evaluated on a real-time control platform (TMS320F28379D). The measured single-cycle execution time of the WNN-DITC is 53µs, compared to 43µs for the conventional DITC, resulting in a marginal increase of approximately 23% in the overall computational load relative to the conventional implementation.

## Conclusions

This article presented a novel DITC scheme for SRMs using an adaptive WNN; thereby addressing the limitations associated with the conventional DITC scheme. The proposed WNN is used to dynamically online compensate the reference torque signal; the network parameters, including weights, translation, and dilation of wavelet functions are optimized once offline using the EO algorithm; then integrated into the developed control technique for a dynamic real-time torque control for SRMs. The superiority of proposed DITC-WNN is validated experimentally, based on a 12/8 SRM prototype, compared conventional DITC. The proposed control features a simple structure with easy implementations, making it a powerful candidate with high capability of generalization; it shows a superior performance meeting the requirements of an EV, providing significant reduction of torque ripples over extended speed ranges.

## Data Availability

The datasets used and/or analyzed in the current study are available from the corresponding author upon reasonable request.
